# Metabolomic analysis reveals altered metabolic pathways in a rat model of gastric carcinogenesis

**DOI:** 10.18632/oncotarget.11049

**Published:** 2016-08-04

**Authors:** Jinping Gu, Xiaomin Hu, Wei Shao, Tianhai Ji, Wensheng Yang, Huiqin Zhuo, Zeyu Jin, Huiying Huang, Jiacheng Chen, Caihua Huang, Donghai Lin

**Affiliations:** ^1^ High-field NMR Research Center, MOE Key Laboratory of Spectrochemical Analysis & Instrumentation, Department of Chemical Biology, College of Chemistry and Chemical Engineering, Xiamen University, Xiamen, P.R. China; ^2^ Department of Physical Education, Xiamen University of Technology, Xiamen, P.R. China; ^3^ Department of Pathology, The Affiliated Chenggong Hospital, Xiamen University, Xiamen, P.R. China; ^4^ Department of Gastrointestinal Surgery, The Affiliated Zhongshan Hospital, Xiamen University, Xiamen, P.R. China; ^5^ State Key Laboratory of Cellular Stress Biology, Innovation Center for Cell Signaling Network, School of Life Sciences, Xiamen University, Xiamen, P.R. China

**Keywords:** gastric carcinogenesis, metabolic network, metabolic pathways, metabolomics, NMR

## Abstract

Gastric cancer (GC) is one of the most malignant tumors with a poor prognosis. Alterations in metabolic pathways are inextricably linked to GC progression. However, the underlying molecular mechanisms remain elusive. We performed NMR-based metabolomic analysis of sera derived from a rat model of gastric carcinogenesis, revealed significantly altered metabolic pathways correlated with the progression of gastric carcinogenesis. Rats were histologically classified into four pathological groups (gastritis, GS; low-grade gastric dysplasia, LGD; high-grade gastric dysplasia, HGD; GC) and the normal control group (CON). The metabolic profiles of the five groups were clearly distinguished from each other. Furthermore, significant inter-metabolite correlations were extracted and used to reconstruct perturbed metabolic networks associated with the four pathological stages compared with the normal stage. Then, significantly altered metabolic pathways were identified by pathway analysis. Our results showed that oxidative stress-related metabolic pathways, choline phosphorylation and fatty acid degradation were continually disturbed during gastric carcinogenesis. Moreover, amino acid metabolism was perturbed dramatically in gastric dysplasia and GC. The GC stage showed more changed metabolite levels and more altered metabolic pathways. Two activated pathways (glycolysis; glycine, serine and threonine metabolism) substantially contributed to the metabolic alterations in GC. These results lay the basis for addressing the molecular mechanisms underlying gastric carcinogenesis and extend our understanding of GC progression.

## INTRODUCTION

Gastric cancer (GC), one of the most prevalent and deadly forms of cancers worldwide, is common in East Asia, especially in Japan and China [1]. Gastric carcinogenesis is a multistep process, in which gastric mucosa undergoes a series of changes resulting in gastritis, atrophy, intestinal metaplasia, and atypical hyperplasia, before developing into GC [2]. Gastric carcinogenesis is also a multifactorial process, specially correlated with the interaction between the host factors, *H. pylori* infection and environmental factors such as diet [3]. Repeated infection and inflammations have been regarded as primary causes of gastric carcinogenesis [4].

Gastric tumors differ from their normal counterparts in many ways, including the increased cell proliferation, cell differentiation and turnover of nutrients, which might result from aberrant metabolisms in gastric mucosa cells. As a potent analysis method, metabolic profiling is extensively used to address the alterations in both metabolic profiles and metabolite levels associated with gastric carcinogenesis. It is also used to exploit the early diagnostic approach for GC [5–7]. Miyagi *et al.* analyzed markedly altered metabolite levels in plasma derived from GC patients with high performance liquid chromatography-mass spectra (HPLC-MS) [5]. Ikeda *et al.* performed metabolic profiling on sera derived from GC patients with gas chromatography-mass spectrometry (GC-MS), and found that both 3-hydropropionic acid and pyruvate made significant contributions to discriminate the metabolic profile of GC patients from that of healthy subjects [6]. These works showed that the levels of some metabolites were changed in both gastric tumors and infected organisms. Furthermore, Yu *et al.* analyzed metabolic alterations in the GC and precancerous stages (chronic superficial gastritis, chronic atrophic gastritis, intestinal metaplasia and gastric dysplasia) with GC-MS [7]. They found that the metabolic phenotype of chronic superficial gastritis was distinctly distinguished from that of GC, while intestinal metaplasia shared similar metabolic phenotype with GC.

As expected, understanding how and why metabolic changes would be greatly helpful for clarifying molecular mechanisms underlying gastric carcinogenesis. Changes of the activities and/or expression levels of a few crucial metabolic enzymes could propagate considerable perturbations into entire cellular functions [8]. Therefore, establishment of perturbed metabolic networks, which link altered metabolites to their associated regulatory enzymes, could enable detail depiction of the metabolic mechanisms underlying gastric carcinogenesis. In the recent years, metabolomic data [9], or integrated with genomic data [10, 11] or transcriptomic data [12], have been used to reconstruct metabolic networks. Based on the concept of correlation spectroscopy, Cloarec *et al.* developed Statistical Total Correlation Spectroscopy (STOCSY) to display the correlations among the intensities of various peaks across the whole sample [13]. STOCSY can offer significant inter-metabolite correlations and metabolic variations in a metabolic network through highlighting simultaneous concentration alterations of metabolites associated with metabolic pathways [9]. However, the biological interpretation of a STOCSY spectrum is complex and not always straightforward. By combining the statistical recoupling of variables (SRV) arithmetic [14] with STOCSY, Blaise *et al*. further developed Recoupled-STOCSY (R-STOCSY) to greatly reduce the dimensionality inherited from the high-resolution bucketing [9]. R-STOCSY allows for identification of meaningful correlations only between distant clusters (metabolic signals). Furthermore, R-STOCSY is validated through measuring the distances between correlated metabolites within the whole metabolic network. Expectedly, the average shortest path length is significantly shorter for the detected correlations in comparison with metabolite couples randomly selected from within the entire Kyoto Encyclopedia of Genes and Genomes (KEGG) metabolic network [9]. However, the unsupervised R-STOCSY is useful only if the targeted perturbation is the dominant factor [15]. By associating orthogonal filters [16], with R-STOCSY, Blaise *et al.* developed orthogonal filtered R-STOCSY (OR-STOCSY) to remove undesirable systemic variation within the data, and identify the inter-metabolite correlations pertinent to minor effects in complex data sets [15]. The supervised OR-STOCSY approach yields correlated metabolites related to perturbations of biology interest, and also enables establishment of perturbed metabolic networks without any a prior knowledge [15].

In the present work, we established a rat model of gastric carcinogenesis induced by N-methyl-N'-nitro-N-nitrosoguanidine (MNNG) and high salt diet, and histologically classified the rats into four pathological groups (gastritis, GS; low-grade gastric dysplasia, LGD; high-grade gastric dysplasia, HGD; GC) and the normal control group (CON). With the procedure shown in Supplementary Figure S1, we performed NMR-based metabolomic analysis of the sera derived from the four pathologic groups of rats (MODEL rats) and CON rats to systematically address the metabolic profiles of the five groups, and also conducted the OR-STOCSY analysis to identify inter-metabolite correlations and extract correlated metabolites, then reconstructed perturbed metabolic networks associated with the four pathological stages in comparison with the normal stage through calculating the shortest path lengths among the correlated metabolites [17]. Thereafter, we identified significantly altered metabolic pathways according to pathway impact values from the pathway topology analysis [18]. Our results may lay the substantial basis for clarifying the molecular mechanisms underlying gastric carcinogenesis.

## RESULTS

### The rat model of gastric carcinogenesis

**Figure 1:**
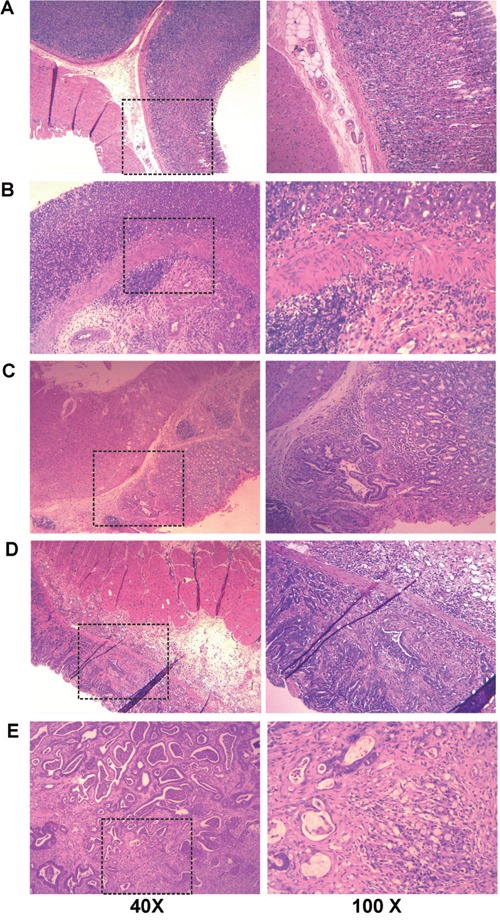
Representative histology images from gastric biopsies for normal rats (CON) and rats in four typical pathological stages (MODEL) The tissue sections were stained with hematoxylin-eosin and observed under the 40×microscope (left column) and the 100×microscope (right column). **A.** CON; **B.** GS; **C.** LGD; **D.** HGD; **E.** GC.

Totally, 128 rats were used for metabolic profiling, including 52 MODEL rats and 32 CON rats, while 44 MODEL rats were lost due to accidental death. None of the CON rats was death in accident. The MODEL rats had lesions only on the bottom of stomach, which were similar to those reported in the references [19–21]. All gastric tissues were embedded in paraffin and sequentially cut for histologic examination. Each tissue section was scored by at two clinical pathologists independently blinded to the histologic examination. According to the updated Sydney system [22] and the Padova International Classification [23], we classified the MODEL rats into four pathologic groups: GS, LGD, HGD and GC (Table 1). The typical images of pathologic histology of gastric tissues are shown in Figure 1. The gastric mucosa of CON rat was normal without atypia (Figure 1A), while that of GS rats showed the infiltration of inflammatory cells (Figure 1B). Compared with normal glands, the dysplastic glands in the LGD rats were lined by crowded elongated cells with large, hyperchromatic nuclei (Figure 1C). In HGD rats, the nuclei of dysplastic glands were more plump and larger than those in LGD rats, the tubular structures of dysplastic glands exhibited branching and folding (Figure 1D). In GC rats, the gastric glands were hardly observed in the gastric mucosa, the cells of glands invaded the gastric wall, infiltrated the muscularis mucosae and submucosa (Figure 1E).

**Table 1:** Experimental design for establishing the rat model of gastric carcinogenesis

Group	Number of the rats	Age/week
control rats (CON)	32	10-40
model rats (MODEL)	52	
GS	11	10-15
LGD	15	16-35
HGD	15	16-42
GC	11	36-51

Four typical pathological stages (GS, LGD, HGD, GC) were identified based on histopathologic examinations and the updated Sydney system, the Padova International Classification.

### Resonance assignments of metabolites and metabolic correlations

**Figure 2:**
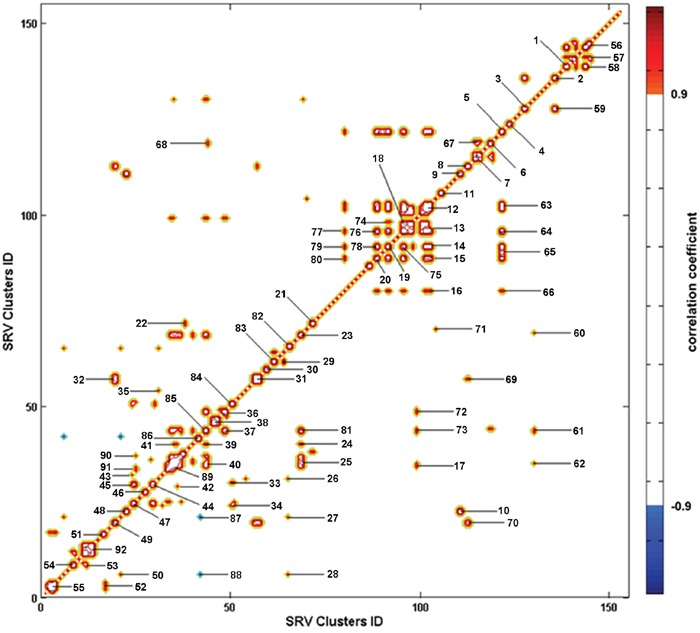
Pseudo-2D R-STOCSY spectrum based on the SRV clusters derived from the NMR data set of MODEL and CON rats Totally, 92 significant metabolic correlations between the clusters were identified with a correlation threshold of 0.9 (Supplementary Table S1 for intra-metabolite correlations, Supplementary Table S2 for inter-metabolite correlations). The degree of metabolic correlation is color-coded (positive correlation in red, negative correlation in blue).

Typica1 1D ^1^H Carr-Purcell-Meiboom-Gill (CPMG) spectra of the sera from CON and MODEL rats are shown in Supplementary Figure S2. Contours in the pseudo-2D R-STOCSY spectrum (Figure 2) represent significant metabolic correlations between the SRV clusters derived from NMR spectral data (intra-metabolite correlations, Supplementary Table S1; inter-metabolite correlations, Supplementary Table S2). The intra-metabolite correlations could aid in assignments of metabolite resonances, while the inter-metabolite correlations enabled identification of significant metabolites involved in the same metabolic pathway whose concentrations are interdependent or under some common regulatory mechanisms. Notably, the R-STOCSY analysis could potentially identify metabolic correlations even for weak NMR signals which are usually missed in a traditional analysis of NMR spectra [9].

We assigned metabolite resonances appearing in 1D ^1^H CPMG spectra based on a combination of literatures [24, 25], Human Metabolome Database (HMDB, www.hmdb.ca/), 2D ^1^H-^1^H Total Correlated Spectroscopy (TOCSY) spectra (Supplementary Figure S3) and pseudo-2D R-STOCSY spectrum (Figure 2). The spin systems of the assigned metabolites were confirmed by ^1^H-^1^H TOCSY spectra. The NMR spectra were mostly dominated by the signals from amino acids and carboxylic acids. We also assigned the intra-metabolite correlations appearing in the R-STOCSY spectrum (Supplementary Table S1).

### Metabolic profiling of the rat model

#### Principal component analysis (PCA)

**Figure 3:**
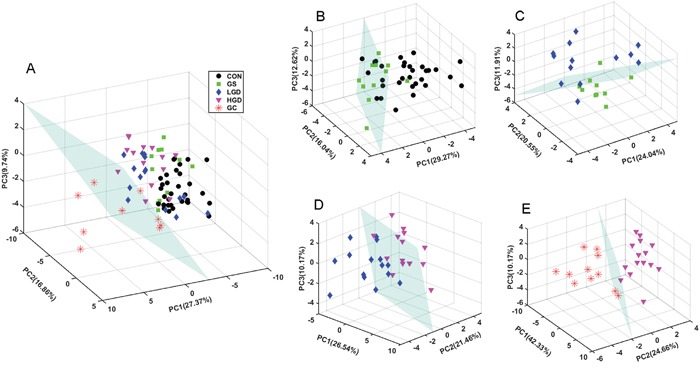
PCA scores plots of SRV clusters derived from NMR spectra of the sera **A.** MODEL rats vs. CON rats; **B.** GS rats vs. CON rats; **C.** LGD rats vs. GS rats; **D.** HGD rats vs. LGD rats; **E.** GC rats vs. HGD rats.

To obtain a comprehensive comparison of metabolic profiles among the five groups of rats, PCA was performed on the data of SRV clusters. PCA scores plots with the first three principal components (PC1, PC2 and PC3) are shown in Figure 3. The metabolic profile of GC displays a clear separation from those of the other groups (CON, GS, LGD and HGD) which are not distinctly distinguishable from one another with partial overlap (Figure 3A). Furthermore, we also conducted pairwise PCA for the five groups (Figure 3B, 3C, 3D, 3E), aiming to assess the changes of metabolic profiles associated with the four pathological stages. We observed a tendency of GS rats being discriminated from CON rats, although some samples could not be clearly separated (Figure 3B). A similar result was obtained from the comparison of metabolic profiles between LGD and GS rats (Figure 3C). The metabolic profile of HGD rats is clearly distinguished from that of LGD rats (Figure 3D). Interestingly, GC rats show a distinctly different metabolic profile from HGD rats without any overlap (Figure 3E).

Notably, alterations in the diet used to establish this model potentially contributed to the metabolic changes in gastric carcinogenesis. To evaluate the unexpected influential effect of the diet, we re-performed PCA by adding serum samples derived from four experimental rats. They were fed with the same diet as the Model rats. We checked the pathological states of the rats every one week. The four rats were not diagnosed to be on either the GS state or other pathological states. The PCA scores plot (Supplementary Figure S4) demonstrates that metabolic profiles of the four added samples are not distinctly distinguished from those of the CON samples. Therefore, it could be expected that the metabolic changes of the MODEL rats mostly came from GC development rather than the diet influence.

#### Partial least squares to latent structure with discriminant analysis (PLS-DA)

The pairwise PLS-DA models and their corresponding response permutation tests (RPTs) were used to explore the differences of metabolic profiles among the five groups. The PLS-DA scores plots show clear separation of GS rats from CON rats, LGD rats from GS rats, HGD rats from LGD rats, GC rats from HGD rats (Supplementary Figure S5). The validation plots of the corresponding RPTs confirm that these classifications are reliable (Supplementary Figure S6). In addition, the PLS-DA scores plots (Supplementary Figure S7) illustrate that almost all the GS, LGD, HGD and GC rats are separated clearly from CON rats, with only one GS rat and two LGD rats are misclassified in the scores plots (Supplementary Figure S7A, S7B). In general, the metabolic profiles of MODEL rats are distinguishable from that of CON rats. The validation plots of the corresponding RPTs indicate that these classifications are not over-fitting (Supplementary Figure S8).

#### Orthogonal projection on latent structure with discriminant analysis (OPLS-DA)

**Figure 4:**
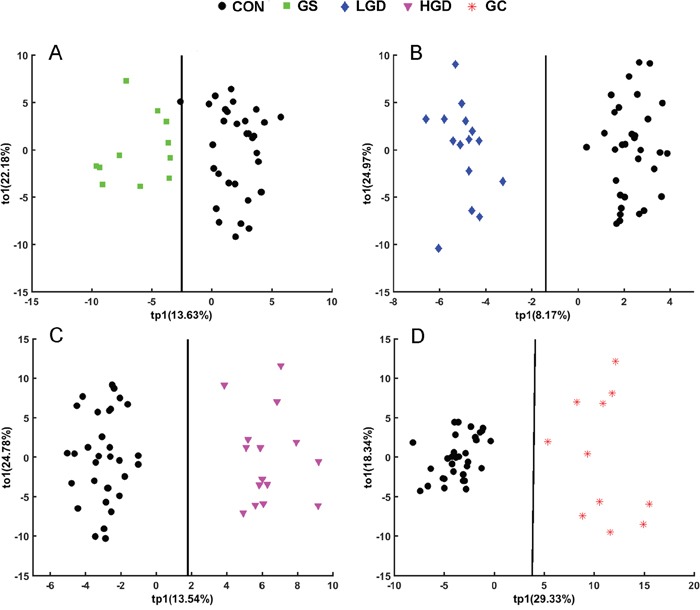
OPLS-DA scores plots of SRV clusters data derived from NMR spectra of the sera showing clear separation of GS rats **A.** LGD rats **B.** HGD rats **C.** GC rats **D.** from CON rats.

The OPLS-DA analysis was conducted to highlight the metabolic alterations of MODEL rats compared with CON rats. The application of the orthogonal filter in OPLS-DA has the tremendous advantage to target and isolate the metabolic variations corresponding to a specific factor (such as pathological effect), even if the amplitude affected by the pathological status is small. The OPLS-DA scores plots are shown in Figure 4 with one predictive principal component (tp1) and one orthogonal component (to1). The linear classifier boundaries exhibit the clear separation of GS, LGD, HGD, GC rats from CON rats (Figure 4).

**Figure 5:**
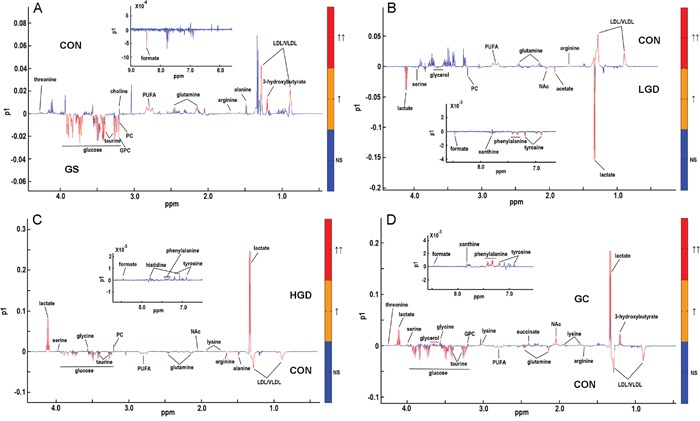
OPLS-DA loading plots used to identify differential metabolites significantly responsible for distinguishing different pathological groups **A.** GS rats vs. CON rats; **B.** LGD rats vs. CON rats; **C.** HGD rats vs. CON rats; **D.** GC rats vs CON rats. The red color indicates that the variables are very significant (|r| > 0.389 in A and D or |r| > 0.372 in B and C; VIP > 1); orange indicates that the variables are significant (0.301 <|r| < 0.389 in A and D or 0.288 <|r| < 0.372 in B and C; VIP > 1); blue indicates that the variables are insignificant (NS).

The corresponding OPLS-DA loading plots were used to identify differential metabolites significantly responsible for the class separation, based on the first predictive principal component (Figure 5). Detailed information of the differential metabolites is shown in Supplementary Tables S3-S6. Totally, 13, 14, 17 and 20 differential metabolites were identified from the OPLS-DA analyses of GS rats vs. CON rats (Figure 5A, Supplementary Table S3, 5 increased, 8 decreased), LGD rats vs. CON rats (Figure 5B, Supplementary Table S4, 10 increased, 4 decreased), HGD rats vs. CON rats (Figure 5C, Supplementary Table S5, 10 increased, 7 decreased), GC rats vs. CON rats (Figure 5D, Supplementary Table S6, 14 increased, 6 decreased), respectively.

Summarily, the PCA, PLS-DA and OPLS-DA scores plots demonstrate that the metabolic profiles are clearly discriminated among the five groups of rats. The OPLS-DA loading plots illustrate that specific sets of differential metabolites are related to different pathological stages. These results indicate the significant pathological effects on the metabolic phenotypes of the sera, which lay the base for the following OR-STOCSY analysis.

### Changed metabolite levels in the four pathological stages

To quantitatively assess the changes in metabolite levels, we calculated relative integrals of the assigned metabolites based on the 1D ^1^H CPMG spectra of the sera derived from CON and MODEL rats. The means and standard errors of relative integrals are shown in Table 2. We then performed multiple comparisons of metabolite levels for the five groups of rats by one-way analysis of variance (ANOVA) followed by the Tukey's multiple comparisons test (Table 2). The results were almost consistent with those from OPLS-DA (Figure 5, Supplementary Tables S3-S6). Only two inconsistencies were found, both regarding the metabolite level comparison of GC rats with CON rats (Figure 5D, Supplementary Table S6, Table 2):, succinate exhibited a significantly increased level identified from OPLS-DA with an insignificantly increased level indicated by ANOVA; asparagine displayed a relative stable level identified from OPLS-DA with a significantly increased level indicated by ANOVA. These results revealed that different pathological stages were related to different changes in metabolite levels. These significantly altered metabolites were mostly involved in four crucial metabolisms: amino acid metabolism, fatty acid metabolism, carbohydrate metabolism and nucleic acid metabolism (Table 2). Note that several metabolites were not involved in the four metabolisms, which are shown as the “other metabolites” in Table 2.

**Table 2:** Comparison of metabolite levels in the five groups of the rats based on relative integrals calculated from the ^1^H NMR spectra of the sera

	Tukey's multiple comparisons test							Mean ± Standard error					One-way ANOVA	
	GS vs.	LGD vs.	HGD vs.	GC vs.	LGD vs.	HGD vs.	GC vs.	CON	GS	LGD	HGD	GC	F	P
	CON	CON	CON	CON	GS	LGD	HGD							
AMINO ACID METABOLISM														
arginine	**	***	***	***	*	NS	NS	0.484 ± 0.038	0.372 ± 0.041	0.290 ± 0.066	0.266 ± 0.048	0.290 ± 0.085	15.09	3.74e-9
glutamine	*	**	***	*	NS	NS	NS	0.406 ± 0.020	0.347± 0.039	0.336 ± 0.036	0.312 ± 0.025	0.345 ± 0.045	3.84	0.031
serine	NS	*	**	**	NS	NS	NS	0.072 ± 0.003	0.074± 0.004	0.081 ± 0.005	0.083 ± 0.005	0.09 ± 0.005	8.84	6.28e-6
tyrosine	NS	**	*	***	NS	NS	NS	0.024 ± 0.002	0.029 ± 0.005	0.031 ± 0.004	0.031 ± 0.005	0.032 ±0.002	4.26	0.004
phenylalanine	NS	**	**	***	**	*	***	0.017 ± 0.005	0.021 ± 0.011	0.032 ± 0.009	0.026 ± 0.009	0.056 ± 0.007	11.30	2.90e-7
glycine	NS	NS	*	**	NS	NS	NS	0.284 ± 0.012	0.280 ± 0.032	0.310 ± 0.028	0.320 ± 0.028	0.364 ± 0.037	4.65	0.002
lysine	NS	NS	*	***	NS	NS	***	0.269 ± 0.014	0.282 ± 0.036	0.301 ± 0.035	0.305 ± 0.030	0.499 ± 0.086	17.38	3.38e-10
threonine	*	NS	NS	***	NS	NS	***	0.214 ± 0.009	0.195 ± 0.018	0.202 ± 0.011	0.201 ± 0.019	0.283 ± 0.024	13.19	3.14e-8
alanine	*	NS	*	NS	NS	NS	*	0.302 ± 0.017	0.267 ± 0.029	0.286 ± 0.038	0.240 ± 0.043	0.304 ± 0.052	3.70	0.008
asparagine	NS	NS	NS	**	NS	NS	*	0.056 ±0.005	0.059 ± 0.008	0.064 ± 0.007	0.059 ± 0.006	0.079 ± 0.018	4.19	0.004
histidine	NS	NS	**	NS	NS	NS	NS	0.024 ± 0.002	0.026 ± 0.003	0.027 ± 0.003	0.029 ± 0.003	0.027 ± 0.004	3.84	0.037
leucine	NS	NS	NS	NS	NS	NS	NS	0.235 ± 0.008	0.222 ± 0.023	0.228 ± 0.014	0.221 ± 0.013	0.216 ± 0.030	0.93	0.451
isoleucine	NS	NS	NS	NS	NS	NS	NS	0.100 ± 0.006	0.097 ± 0.013	0.105 ± 0.007	0.096 ± 0.007	0.098 ± 0.023	0.65	0.630
valine	NS	NS	NS	NS	NS	NS	NS	0.170 ± 0.011	0.167 ± 0.023	0.177 ± 0.011	0.169 ± 0.012	0.139 ± 0.033	0.71	0.536
isobutyrate	NS	NS	NS	NS	NS	NS	NS	0.011 ±0.009	0.010 ± 0.002	0.012 ± 0.001	0.011 ± 0.002	0.012 ± 0.002	1.86	0.126
glutamate	NS	NS	NS	NS	NS	NS	NS	0.279 ± 0.018	0.270 ± 0.019	0.275 ± 0.021	0.302 ± 0.033	0.274 ± 0.033	0.84	0.501
aspartate	NS	NS	NS	NS	NS	NS	NS	0.325 ± 0.015	0.315 ± 0.030	0.348 ± 0.029	0.342 ± 0.013	0.317 ± 0.037	1.15	0.339
FATTY ACID METABOLISM														
LDL/VLDL	**	***	***	***	*	*	**	6.711 ± 0.318	5.984 ± 0.412	5.550 ± 0.712	4.922 ± 0.523	4.156 ± 1.059	12.52	6.80e-8
PUFA	****	***	***	***	NS	*	*	2.561 ± 0.114	1.766 ± 0.185	1.741 ± 0.294	1.371 ± 0.172	1.764 ± 0.563	21.42	6.62e-12
3-hydroxybutyrate	*	NS	NS	**	*	NS	**	0.321 ± 0.055	0.225 ± 0.089	0.378 ± 0.125	0.285 ± 0.144	0.601 ± 0.156	6.08	0.021
glycerol	NS	*	NS	***	NS	NS	***	0.556 ± 0.018	0.562 ± 0.053	0.599 ± 0.023	0.584 ± 0.031	0.729 ± 0.091	9.49	2.72e-6
acetate	NS	*	NS	NS	NS	NS	NS	0.126 ± 0.008	0.134 ± 0.012	0.147 ± 0.013	0.134 ± 0.009	0.136 ± 0.038	3.36	0.026
CARBOHYDRATE METABOLISM														
lactate	NS	***	***	***	***	**	***	3.641 ± 0.171	3.939 ± 0.312	4.406 ± 0.389	4.874 ± 0.273	6.107 ± 0.566	48.44	2.70e-20
glucose	*	NS	*	*	*	*	NS	1.619 ± 0.117	1.837 ± 0.098	1.555 ± 0.201	1.285 ± 0.166	1.249 ± 0.247	5.98	3.01e-4
succinate	NS	NS	NS	NS	NS	NS	NS	0.019 ± 0.003	0.015 ± 0.004	0.023 ± 0.005	0.020 ± 0.006	0.028 ± 0.006	1.97	0.108
citrate	NS	NS	NS	NS	NS	NS	NS	0.107 ± 0.007	0.099 ± 0.015	0.116 ± 0.015	0.115 ± 0.008	0.088 ± 0.020	2.20	0.077
fumarate	NS	NS	NS	NS	NS	NS	NS	0.0013±0.0007	0.0006±0.0005	0.0006±0.0004	0.0009±0.0005	0.0007±0.0005	1.25	0.413
NUCLEIC ACID METABOLISM														
xanthine	NS	***	NS	***	NS	NS	*	0.076 ± 0.007	0.081 ± 0.011	0.093 ± 0.007	0.086 ± 0.008	0.118 ± 0.020	7.13	6.13e-5
OTHER METABOLITES														
formate	***	***	***	***	NS	NS	**	0.009 ± 0.001	0.013 ± 0.003	0.013 ± 0.002	0.015 ± 0.002	0.022 ± 0.004	15.73	1.89e-9
PC	***	***	***	NS	NS	*	***	0.045 ±0.004	0.06 ± 0.004	0.058 ± 0.007	0.069 ± 0.007	0.046 ± 0.007	12.58	6.32e-8
α-acid glycoprotein	NS	*	*	***	*	NS	***	2.234 ± 0.075	2.228 ± 0.209	2.638 ± 0.133	2.615 ± 0.182	3.193 ± 0.597	8.62	8.37e-6
taurine	**	NS	*	*	NS	*	NS	0.218 ± 0.013	0.253 ± 0.011	0.225 ± 0.030	0.182 ± 0.024	0.170 ± 0.037	5.90	3.38e-4
GPC	**	NS	NS	***	NS	NS	***	0.064 ± 0.002	0.075 ± 0.005	0.068 ± 0.006	0.071 ± 0.007	0.085 ± 0.006	8.24	1.37e-5
choline	*	NS	NS	NS	NS	NS	NS	0.021 ± 0.001	0.017 ± 0.002	0.019 ± 0.002	0.018 ± 0.002	0.022 ± 0.004	2.29	0.048
creatine	NS	NS	NS	NS	NS	NS	NS	0.205 ± 0.022	0.161 ± 0.037	0.194 ± 0.031	0.171 ± 0.037	0.213 ± 0.017	3.20	0.067

Note: Symbols ***, **, *, NS mean differences between A and B were highly significant (p<0.001), very significant (p<0.01), significant (p<0.05), insignificant (p>0.05), respectively. Red and blue colors denote that the difference is positive (i.e. A was increased compared to B) and negative, respectively.

Furthermore, a heatmap plot was produced to clearly display the metabolites level differences among the five groups (Supplementary Figure S9). According to the standardization protocol of metabolite levels, the relative integral of each metabolite was centered to have a mean of zero and scaled to have a standard deviation of one.

#### Amino acid metabolism

Both arginine and glutamine were significantly decreased in MODEL rats; seine, tyrosine, and phenylalanine were markedly increased in LGD, HGD, GC rats; glycine and lysine showed higher levels in HGD and GC rats; threonine was decreased in GS rats, and increased dramatically in GC rats; alanine was decreased in GS and HGD rats; histidine was increased in HGD rats; asparagine was significantly enhanced in GC rats. Notably, branch chain amino acids (leucine, isoleucine and valine), isobutyrate, glutamate and aspartate did not show distinctly changed levels in MODEL rats.

#### Fatty acid metabolism

Both LDL/VLDL and polyunsaturated fatty acid (PUFA) were dramatically decreased in MODEL rats; 3-hydroxybutyrate exhibited fluctuated levels in MODEL rats, with a decreased level in GS rats and a significantly increased level in GC rats; glycerol was increased in LGD and GC rats, exhibiting the highest level in GC rats; acetate was slightly increased in LGD rats.

#### Carbohydrate metabolism

Lactate was markedly increased in LGD, HGD, GC rats; glucose displayed fluctuated levels in MODEL rats, with an increased level in GS rats and decreased levels in HGD and GC rats; three primary metabolites (succinate, citrate and fumarate) involved in TCA cycle did not show distinctly changed levels in MODEL rats.

#### Nucleic acid metabolism

Compared with that in CON rats, xanthine showed high levels in LGD and GC rats, approaching the highest level in GC rats.

#### Other metabolites

Formate was markedly enhanced in MODEL rats; phosphocholine (PC) was remarkably increased in GS, LGD, HDG rats; α-acid glycoprotein was increased in LGD, HGD and GC rats, displaying the highest level in GC rats; taurine exhibited fluctuated levels in MODEL rats, with a dramatically increased level in GS rats and decreased levels in HGD and GC rats; Glycerophosphocholine (GPC) was greatly increased in GS and GC rats with the highest level in GC rats, which did not show distinctly changed levels in LGD, HGD and GC rats; choline was slightly decreased in GS rats with relative stable levels in LGD, HGD, GC rats; creatine did not exhibit significantly altered levels in MODEL rats.

### Perturbed metabolic networks and significantly altered metabolic pathways during gastric carcinogenesis

**Figure 6:**
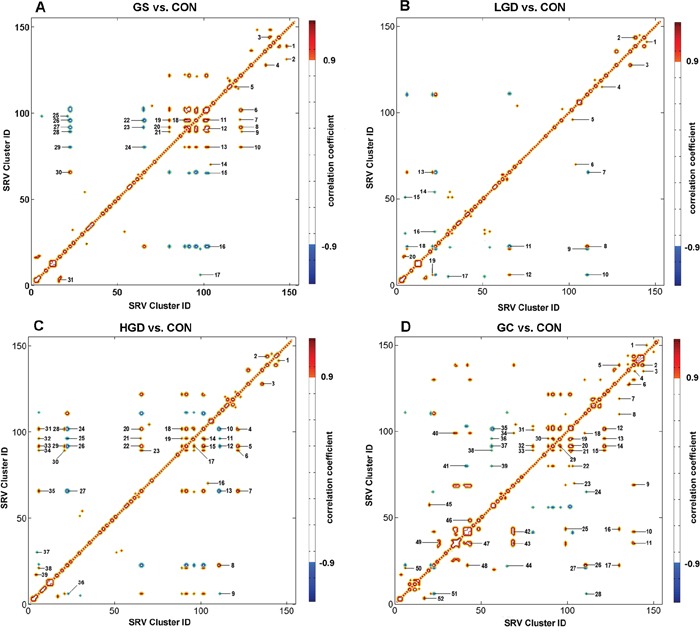
Pseudo-2D OR-STOCSY spectra based on SRV clusters derived from the NMR data set of the sera Significant inter-metabolite correlations between clusters were identified from the OR-STOCSY analysis for **A.** GS rats vs. CON rats; **B.** LGD rats vs. CON rats; **C.** HGD vs. CON rats; **D.** GC rats vs. CON rats (Supplementary Tables S7-S10). The degree of metabolic correlation is color-coded (red for positive correlation, blue for negative correlation) with a correlation threshold of 0.9.

To exploit the molecular mechanisms underlying gastric carcinogenesis, we performed the OR-STOCSY analysis to identify significant inter-metabolite correlations with a correlation threshold of 0.9, and reconstructed perturbed metabolic networks associated with the four pathological stages in comparison with the normal stage. Totally, 31, 20, 39, 52 inter-metabolite correlations were extracted from the OR-STOCSY analyses of GS vs. CON (Figure 6A; Supplementary Table S7), LGD vs. CON (Figure 6B; Supplementary Table S8), HGD vs. CON (Figure 6C; Supplementary Table S9), GC vs. CON (Figure 6D; Supplementary Table S10), respectively. From these inter-metabolite correlations, 18, 20, 20, 27 metabolites were identified for GS, LGD, HGD and GC stages, respectively.

**Figure 7:**
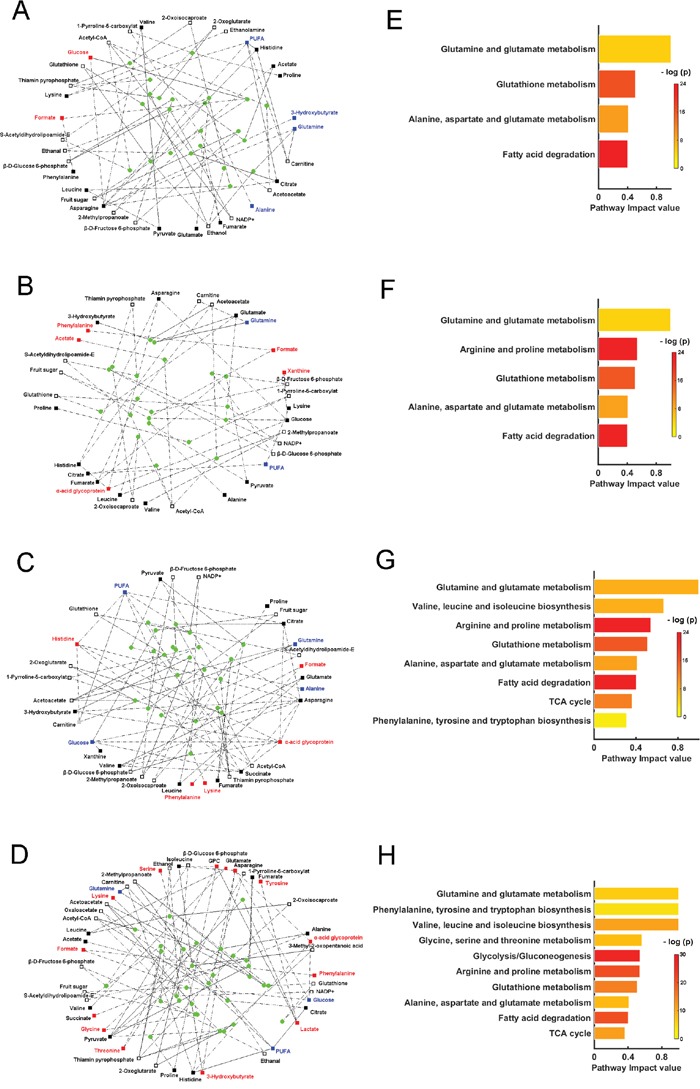
Perturbed metabolic networks and significantly altered metabolic pathways associated with four typical pathological stages of gastric carcinogenesis compared with the normal stage **A, E.** GS vs. CON; **B, F.** LGD vs. CON; **C, G.** HGD vs. CON; **D, H.** GC vs. CON. Filled squares are the metabolites with significant inter-metabolite correlations identified from OR-STOCSY spectra, which were used to calculate the shortest path lengths and reconstruct perturbed metabolic networks. The red, blue and black filled squares denote significantly increased (p < 0.05), significantly decreased levels (p < 0.05), and insignificantly changed levels (p > 0.05) in MODEL rats compared with those in CON rats, respectively. Unfilled squares are NMR-invisible metabolites and filled circles are significant enzymes, both extracted from the calculation of the shortest path lengths. Significantly altered metabolic pathways were identified based on pathway impact values calculated from the pathway topology analysis with a threshold of 0.3. The p value in the significantly altered metabolic pathways (right column) was calculated from the enrichment analysis.

Through calculating the shortest path lengths among the correlated metabolites [17], we extracted metabolites, enzymes (Supplementary Tables S11-S14) and metabolite-enzyme relations from the homebuilt biograph object file (described in the Methods section) to reconstruct the perturbed metabolic networks associated with the four pathological stages (Figure 7A-7D). The one-way ANOVA analysis described above together with OPLS-DA, indicated the changes of the metabolite levels in MODEL rats compared to those in CON rats (Tables 2, Supplementary S3-S6). The significantly increased, significantly decreased and insignificantly changed metabolites are displayed as red, blue and black filled squares (Figure 7A-7D). NMR-invisible metabolites are shown as unfilled squares. From the reconstructed metabolic networks, we identified significantly altered metabolic pathways according to pathway impact values from the pathway topology analysis with a threshold of 0.3 [18]. Compared with the CON stage, 4, 5, 8, 10 significantly altered metabolic pathways were identified for GS, LGD, HGD and GC stages, respectively (Figure 7E-7H), which provide a mechanistic understanding of gastric carcinogenesis.

#### The GS stage

Totally, 35 extracted metabolites (18 correlated metabolites, 17 NMR-invisible metabolites), 23 enzymes and 98 metabolite-enzyme relations were used to reconstruct the perturbed metabolic network associated with GS (Figure 7A, Supplementary Table S10). Four significantly altered metabolic pathways were identified, including glutamate and glutamine metabolism; glutathione (GSH) metabolism; alanine, aspartate and glutamate metabolism; fatty acid degradation (Figure 7E). Both glutamate and glutamine metabolism and GSH metabolism involved five metabolites (glutathione, NADP+, α-ketoglutarate (α-KG), glutamate, glutamine) and seven enzymes, which played important roles in oxidative stress.

#### The LGD stage

Totally, 33 extracted metabolites (20 correlated metabolites, 13 NMR-invisible metabolites), 20 enzymes and 60 metabolite-enzyme relations were used to reconstruct the perturbed metabolic network associated with LGD (Figure 7B, Supplementary Table S11). Five significantly altered metabolic pathways were identified, including glutamate and glutamine metabolism; arginine and proline metabolism; GSH metabolism; alanine, aspartate and glutamate metabolism; fatty acid degradation (Figure 7F). Besides the four metabolic pathways identified in GS, one extra metabolic pathway (arginine and proline metabolism) was markedly changed in LGD, involving three metabolites (lysine, 1-pyrroline-5-carboxylat and proline). Note that as an immune-modulating molecule [26], α-acid glycoprotein became involved in the perturbed metabolic network associated with LGD, exhibiting the correlations with three metabolites (glucose, asparagine, histidine) as shown in Figure 7B.

#### The HGD stage

Totally, 34 extracted metabolites (20 correlated metabolites, 14 NMR-invisible metabolites), 22 enzymes and 107 metabolite-enzyme relations were used to reconstruct the perturbed metabolic network associated with HGD (Figure 7C, Supplementary Table S12). Eight significantly altered metabolic pathways were identified, including glutamate and glutamine metabolism; valine, leucine and isoleucine biosynthesis; arginine and proline metabolism; GSH metabolism; alanine, aspartate and glutamate metabolism; fatty acid degradation; tricarboxylic acid (TCA) cycle; phenylalanine, tyrosine and tryptophan biosynthesis (Figure 7G). Besides the five metabolic pathways identified in LGD, three extra metabolic pathways were significantly altered in HGD, including valine, leucine and isoleucine biosynthesis; TCA cycle; phenylalanine, tyrosine and tryptophan biosynthesis. As one of the core metabolites in TCA cycle, succinate became involved in the perturbed metabolic network, displaying the correlations with six metabolites (glucose, valine, histidine, PUFA, asparagine, α-acid glycoprotein) as shown in Figure 7C.

#### The GC stage

Totally, 45 extracted metabolites (27 correlated metabolites, 18 NMR-invisible metabolites), 31 enzymes and 125 metabolite-enzyme relations were used to reconstruct the perturbed metabolic network associated with GC (Figure 7D, Supplementary Table S13). Up to ten significantly altered metabolic pathways were identified, including glutamate and glutamine metabolism; phenylalanine, tyrosine and tryptophan biosynthesis; valine, leucine and isoleucine biosynthesis; glycine, serine and threonine metabolism; glycolysis/gluconeogenesis; arginine and proline metabolism; GSH metabolism; alanine, aspartate and glutamate metabolism; fatty acid degradation; TCA cycle (Figure 7H). Notably, succinate was significantly enhanced in GC with insignificantly changed level in the precancerous stages (Supplementary Tables S3-S6). In addition to the eight metabolic pathways identified in HGD, two extra metabolic pathways were distinctly altered in GC, including glycine, serine and threonine metabolism, glycolysis/gluconeogenesis.

## DISCUSSION

Gastric carcinogenesis is a multifactor and multistep process. Previous works have performed metabolic profiling of tissues and sera derived from both GC patients and GC animal models [6, 27, 28], and highlighted the metabolic profiles of GC. However, so for few works have been reported to clarify the globe metabolic alterations associated with GC progression, and reveal the underlying molecular mechanisms.

In the present study, we established a rat model of gastric carcinogenesis, observed distinctly different metabolic profiles and changed metabolite levels linked to gastric carcinogenesis. Based on pseudo-2D OR-STOCSY spectra, we extracted metabolites, enzymes and metabolite-enzyme relations and reconstructed them into perturbed metabolic networks. From the pathological stages-related metabolic networks, we identified significantly altered metabolic pathways and significant metabolites through pathway analysis. To our knowledge, this work for the first time depicted the perturbed metabolic networks associated with the four pathological stages of gastric carcinogenesis.

### Disordered oxidative stress might be responsible for dominant metabolic alterations in gastritis

**Figure 8:**
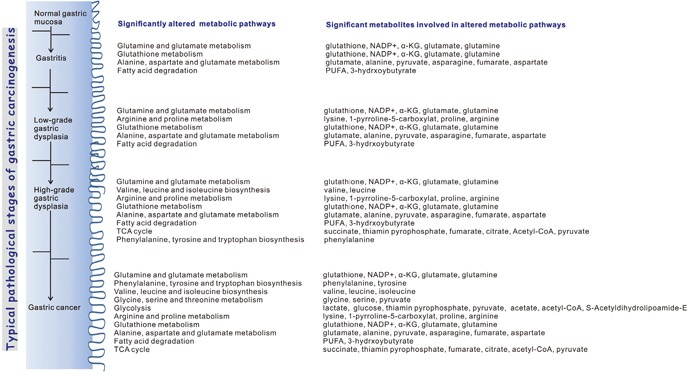
Schematic representation of significantly altered metabolic pathways associated with the four typical pathological stages of gastric carcinogenesis compared with the normal stage

Four significantly altered metabolic pathways in the GS stage include glutamate and glutamine metabolism; GSH metabolism; alanine, aspartate and glutamate metabolism; fatty acid metabolism (Figure 8). These results suggest that oxidative stress might be an early primary event in GS. Chronic inflammation, such as GS, was closely correlated to oxidative stress through generating high concentration of reactive oxygen species (ROS).

Animal models of GS, either *H. pylori*-infected or high salt diet-induced, were mostly associated with increased oxidative DNA damage. Previous works have detected high levels of 8-oxodG (a marker for oxidative DNA damage) in DNA from the tissues of patients with chronic atrophic gastritis and intestinal metaplasia, *H. pylori* infected patients [29], and GC patients as well [30].

The fatty acid metabolism was significantly altered in GS. 3-hydrobutyrate is usually synthesized from acetoacetate as the production of fatty acid degradation. Chronic inflammation has been well established to be a contributor of lipid peroxidation. In this work, the pathway analysis identified an excess fatty acid oxidation in GS. Furthermore, both decreased PUFA and increased 3-hydroxybutyrate were indicative of activated fatty acid degradation in GS. Similar results have been reported by Yoshinori Ito *et al* [31]. They analyzed serum levels of fatty acids by GC-MS, and detected decreased levels of saturated fatty acids when the mice were infected with *H. pylori*.

As an extensively studied metabolic pathway, the GSH metabolism has been found to work as free radical scavengers and promotes DNA damage repairs and play crucial roles in oxidative defense systems. When gastric mucosal damage occurs, shift of the metabolic flux to Pentose phosphate pathway (PPP) would generate a high level of GSH. This would enhance the capability of removing ROS, and thus prevent ROS-induced injury in gastric epithelial cells [32].

In short, enhanced oxidative stress might be a salient metabolic basis for GS which caused the disorders in its relevant metabolic pathways. Moreover, our results demonstrated that oxidative stress was also linked to other pathological stages (LGD, HGD, GC), reflecting an increase risk of oxidative stress in gastric carcinogenesis.

### Amino acids levels were markedly increased during gastric dysplasia

Here we examined the perturbed metabolic networks associated with the two dysplasia stages (LGD, HGD). Both dysplasia stages showed increasing tendencies of amino acid levels, especially in HGD. The increased levels of amino acids were frequently observed in patients with gastric diseases, which made significant contributions to distinguish GC from normal subjects [33]. In addition, pathway analysis identified several disturbed amino acid-related pathways in HGD, including phenylalanine, tyrosine and tryptophan biosynthesis, valine, leucine and isoleucine biosynthesis. Pathway analysis also identified that TCA cycle became disordered in HGD, even though the levels of TCA intermediates were not significantly changed. Furthermore, the perturbed metabolic networks exhibit more inter-metabolite correlations between amino acids and TCA intermediates (such as succinate) in HGD than those in GS and LGC stages, These results imply that amino acid metabolism might be correlated with TCA cycle.

Amino acid could enter TCA cycle through being converted into TCA intermediates for generating ATP. Chan *et al.* analyzed metabolomic profiles of GC, and found that both TCA intermediates and amino acids were elevated, probably resulting from anaplerotic reactions [33]. It is well known that cancerous cells could enhance anaplerotic reactions. Our work observed increased levels of phenylalanine, tyrosine, lysine and serine were increased in HGD. These amino acids could be converted into fumarate and α-KG [34]. BCAAs are the most frequently identified amino acids in GC, and play critical roles in tumor growth and survival by coordinating cellular bioenergetics and biosynthesis through TCA cycle [35].

Contrary to other amino acids, glutamine showed significantly decreased levels in the four pathological stages, approaching the lowest level in HGD. It is thus suggested that glutamine, the most abundant amino acid in mammals, was utilized as an alternative source of TCA cycle anaplerosis. Furthermore, glutamine always works as an important metabolite consumed by the reductive carboxylation to sustain anabolic processes [36]. The decreased glutamine level might reflect the weakened antioxidant defense system as gastric mucosal damage occurred and GC progressed.

In addition to the four disordered metabolic pathways identified in GS, the arginine and proline metabolism was also significantly altered in the dysplasia stages (Figure 8). This altered metabolism was indicative of the ongoing enhanced oxidative stress in dysplasia as GS. The electrons from the arginine and proline metabolism produced ROS and initiated a variety of downstream effects [37], potentially brought the influence to the nucleotide metabolism and RNA production as reported previously [38]. Liu *et al.* found that the product of proline oxidation, namely 1-pyrroline-5-carboxylat, could be recycled back into proline with redox transfers [39]. The disorder of proline oxidase might increase the cancer risk [40].

Gastric carcinogenesis is a multi-stage nonlinear dynamical process. Our work indicated that both dysplasia-related metabolic pathways and levels of some metabolites were remarkably altered also in GC. These results suggest that gastric dysplasia might be near the critical state, which could be detected by dynamical network biomarkers based on the measured metabolites [41, 42]. These biomarkers could be potentially used for early diagnosis of GC.

Summarily, in gastric dysplasia stages some amino acids were enhanced and the relevant metabolic pathways were activated. These amino acids potentially acted as alternative sources to replenish the pools of metabolic intermediates in TCA cycle, and fit metabolic requirements of cell proliferation [36].

### Disordered glycolysis substantially contributed to metabolic alterations in gastric cancer

The GC stage showed more significant metabolic alterations than the precancerous stages, in which up to ten significantly altered metabolic pathways were identified (Figure 8). In GC, glycolysis/gluconeogenesis became disordered, while lactate and glucose approached the highest level and lowest level, respectively. These results implied that glycolysis was activated in GC (Figure 7H). More metabolites are converted into lactate through glycolysis in tumor cells, which has been known historically as the “Warburg effect”. Enhanced aerobic glycolysis is a unique metabolic feature of cancer, which was observed frequently in metabolomic research of GC [33]. However, it is less consistent whether oxidative phosphorylation pathways were suppressed in gastric cancer cells [33]. Regarding TCA cycle, some previous studies demonstrated increased levels of certain TCA cycle intermediates, such as malate, citrate and fumarate [33], while Cai *et al*. [43] reported several inhibited enzymes in TCA cycle. They performed a combined proteomics and metabolomics profiling on gastric cardia cancer, and detected five stimulated enzymes correlated with glycolysis, as well as five inhibited enzymes involved in TCA cycle and PPP pathways [43].

Compared with those in the precancerous stages, more amino acids-related metabolic pathways were identified, and more amino acids were dramatically increased in GC. The glycine, serine and threonine metabolism was activated with significantly increased levels of the glucogenic amino acids, which were associated with tumorigenesis. Jain *et al.* showed that glycine was closely correlated with the disordered glycolysis, working as a key metabolite in the rapid cell proliferation of tumors [44]. Furthermore, serine was derived from a branch of glycolysis and could be converted to glycine [45]. In addition, serine and glycine biosynthesis might suppress cellular antioxidative capacities and thus supported tumor homeostasis [46]. Similarly to HGD stage, the GC stage showed the disturbed BCAA biosynthesis and phenylalanine, tyrosine and tryptophan biosynthesis.

Although previous works have reported the up-regulated flux through certain amino acids metabolism, the underlying mechanisms remain elusive. The metabolic fate of amino acids is intertwined with various process, including metabolic pathways such as TCA cycle, glycolysis, synthesis of protein, phospholipid and nucleotide, production of intermediates for one-carbon metabolism, and maintenance of cellular osmolality, etc. Given amino acid metabolism is correlated with complicated interaction networks in carcinogenesis, it remains a future challenge to establish a precise link between the altered amino acid metabolisms and cancer progression.

### Choline phosphorylation kept disorder and fatty acid degradation kept stimulated during gastric carcinogenesis

Choline, PC and GPC could transform reciprocally during the choline phosphorylation process [47]. The increased level of total choline (choline, PC and GPC) has been extensively reported in a wide variety of cancers [48]. In this work, we detected markedly increased level of total choline together with the altered levels of PC and GPC during gastric carcinogenesis. This observation was indicative of the disturbance of choline phosphorylation in gastric carcinogenesis. The disturbed choline phosphorylation in cancer was usually accompanied by consequent alterations of choline-containing metabolites [49]. Furthermore, alteration in the levels of choline, PC and GPC could lead to fluctuated fatty acid levels and thereby activated fatty acid degradation [50]. Our work demonstrated that the pathway of fatty acid degradation kept activated during gastric carcinogenesis. Expectedly, the activated fatty acid degradation is a common feature of all cancers, since cellular proliferation requires fatty acids for synthesis of membranes and signing molecules [50].

In addition, the level of α-acid glycoprotein was increased in LGD, HGD and GC stages. As an immunomodulating molecule, α-acid glycoprotein usually participates in the immune regulation process [51]. Tilg *et al.* has demonstrated that α-acid glycoprotein facilitated the secretion of an IL-1 inhibitor by murine macrophages, most probably working as an IL-1 receptor antagonist [52]. Moreover, Nakamura *et al.* showed that monocytes stimulated by inflammatory cytokines, could produce α-acid glycoprotein and thereby promoted the fatty acid metabolism for the cell proliferation [53]. Thus, the increased level of α-acid glycoprotein could not only enhance metabolic alterations, but also stimulate the immune response during gastric carcinogenesis.

### Limitations

Expectedly, the identified metabolic pathways could work as key modules to distinguish the five stages. However, in this work, we only detected the metabolite levels without measurements of the expression levels and activities of regulatory enzymes involved in the reconstructed metabolic networks. An integrated analysis, combining the differential metabolites with their upstream genes or proteins, should be conducted to specifically elucidate significant metabolic regulatory pathways underlying GC progression.

In addition, it should also be noted that the perturbed metabolic networks were mainly reconstructed based on the inter-metabolite correlation analysis, which could not correspond to the causal or direct the associations between molecules or between the pathways. Some analysis methods have been developed based on the direct or causal associations, which could be used to accurately quantify direct associations [54, 55] or distinguish direct dependencies in regulatory networks [56].

In conclusion, we performed NMR-based metabolomic profiling of the sera derived from a rat model of gastric carcinogenesis. We reconstructed the perturbed metabolic networks, identified significantly altered metabolic pathways and significant metabolites with distinctly changed levels for the four pathological stages of gastric carcinogenesis. We found that oxidative stress-related metabolic pathways, choline phosphorylation and fatty acid degradation, were continually disturbed during the progression from GS to GC. The amino acid metabolism was perturbed dramatically during gastric dysplasia and GC. In GC stage, more metabolite levels and more metabolic pathways were significantly altered. Two pathways (glycolysis; glycine, serine and threonine metabolism) became activated in GC. Our results shed light on the molecular mechanisms underlying gastric carcinogenesis, and may be of great benefit to the detailed understanding of the development and progression of GC.

## MATERIALS AND METHODS

### Chemicals and animal diets

NaH_2_PO_4_·2H_2_O and K_2_HPO_4_·3H_2_O (all analytical grade) were purchased from Sinopharm Chemical Reagent Co., Ltd. (Shanghai, China), while analytical grade sodium azide (NaN_3_) was obtained from Sangon Biotech (Shanghai) Co., Ltd. (shanghai, China). N-methyl-N'-nitro-N-nitrosoguanidine (MNNG) was purchased from TCI (Shanghai) Development Co., Ltd (Shanghai, China), while the custom 8% NaCl chow pellets were obtained from Suzhou Shuangshi Laboratory Animal Feed Science Co., Ltd. (Suzhou, China). MNNG was dissolved in water at a concentration of 1 mg/ml and kept in the refrigerator at 4°C. The stock solution of MNNG was diluted to 100 μg/ml by tap water just before use.

### Animal experiments

The male Wistar rats (age 3 weeks) were purchased from Shanghai Experimental Animal Center of the Chinese Academy of Sciences (Shanghai, China). The study was performed in accordance with protocols approved by Xiamen University Animal Ethics Committee. All 128 animals were housed in suspended, wire-bottomed cages in animal quarters at a controlled temperature (20-24°C) and humidity (30-50%), with a 12-hr/12-hr light/dark cycle. Rats were randomly divided into the MODEL group (n=96) and CON (normal control) group (n=32). After one week of habituation, each MODEL rat was given the MNNG solution (100 μg/ml) from a bottle covered with aluminum foil to prevent photolysis of MNNG. The solution was replenished every day. MODEL rats were given chow pellets with 8% NaCl [57], while CON rats were fed standard rodent chow pellets and water. After 40 weeks MODEL rats were given standard rodent chow pellets and water. During the process of establishing the rat model, one or two MODEL rats were randomly sacrificed by exsanguinations under ether anesthesia after 10 weeks. CON rats were randomly sacrificed at the same time points. The blood was drawn to prepare serum samples for NMR spectroscopic analysis. Then the lesion stomach tissues were excised and fixed in 10% formalin for histopathological examination. Primary organs of the rats have also been histopathologically examined to ensure the model specificity. All surviving rats were sacrificed at the end of the experiment (in 51th week).

### Histopathology

The stomach tissues from MODEL and CON rats were fixed in 10% formalin. After dehydrating, the biopsies embedded in wax were sectioned at 5 μm, and stained with hematoxylin and eosin for histopathological examination by light microscopy [22, 23].

### Sample preparation and NMR measurements

The serum samples (300 μL) were thawed on the ice, and mixed with 210 μL of deuterated phosphate buffer (50 mM, pH 7.4). After centrifugation at 12000 g at 4°C for 10 min to remove the precipitates, 500 μL of the supernatants was transferred into a 5 mm NMR tube and analyzed by NMR spectroscopy [58]. 1D ^1^H CPMG spectra of these samples were acquired on a Bruker Avance III 600 MHz spectrometer at 25°C using the pulse sequence [RD-90°-(τ-180°-τ)_n_-ACQ] with water suppression. A fixed total spin-spin relaxation delay of 80ms were used to attenuate broad NMR signals of slowly tumbling molecules with short T_2_ relaxation times, and retain signals of low molecular weight compounds. The spectral width was 12 KHz with an acquisition time per scan of 2.7 s, and a total of 256 transients were collected into 64K data points for each spectrum. For the purpose of resonance assignments, 2D NMR ^1^H-^1^H TOCSY spectra were recorded on selected samples. The acquisition parameters were referred to the literatures [24, 59].

### Data processing and multivariate statistical analysis

The FIDs were multiplied by an exponential function corresponding to a 0.3 Hz line-broadening factor before Fourier transformation. The obtained NMR spectra were manually phased and baseline-corrected by using the Bruker TOPSPIN 2.1 package (Bruker Biospin, Germany) and referenced to the CH_3_ resonance of lactate at 1.33 for serum spectra. Each ^1^H NMR spectrum was segmented into regions of 0.001 ppm and integrated using the MestReNova Version 6.1 (Mestrelab Research S.L, Espain). For the metabolites with highly overlapping peaks, we selected the non-overlapping peaks to accurately calculate the spectral integrals for these metabolites. The spectral region of serum was δ 9.00-0.00, while the data of region δ 5.7-4.6 was set to zero to eliminate distorted baseline from imperfect water saturation. The icoshift algorithm was performed to remove misalignment of NMR signals in MATLAB (Math Works, USA) [60].

Normalization was applied to the total sum of integrated data from each sample (a constant integral of 100 was used), making the data directly comparable with each other [61, 62]. Relative integrals of the identified metabolites (Supplementary Figure S2) were used for comparison among the five groups of rats. Variations were calculated by utilizing one way ANOVA followed by the Tukey's multiple comparisons test in MATLAB. The variables with p < 0.05 were considered as statistically significant.

Furthermore, the SRV arithmetic was utilized to analyze the NMR spectra [14, 63]. SRV is an automated variable size bucketing procedure aiming at the identification of statistically significant metabolic NMR peaks [9]. The effect of SRV is achieved by focusing on the statistical relationships between consecutive variables inherited from the high-resolution bucketing without a priori knowledge. The SRV approach could greatly reduce the dimensionality inherited from the high-resolution bucketing to decrease invalid signals of the metabolite correlations. We performed the SRV arithmetic in MATLAB. The following parameters were used to efficiently recouple variables inherited from the high-resolution bucketing: peak base width at the noise threshold for a resolved weak singlet in the aromatic area of 0.01 ppm (singlet size), bucketing resolution of 0.001 ppm. Totally, 153 SRV clusters were identified in the CPMG spectra, representing 84.42% of the spectral signals (Supplementary Figure S10).

As the following OR-STOCSY analysis was uniquely conducted on the data of the SRV clusters to identify intra/inter-metabolite correlations, PCA was thus performed on the SRV data too for keeping the consistency of data analysis. Using the software SIMCA-P+12.0 (Umetrics, Sweden), we performed PCA to reveal trends, highlight outliers, and show clusters among the observations. The parameters R^2^X(cum) and Q^2^(cum) were used to evaluate the quality of the PCA model. R^2^X(cum) denotes the fraction of the sum of the squares of the explanation of the integral values in the model, while Q^2^(cum) represents the cross-validated explained variation [64]. Based on the PCA scores plots, discriminant planes were calculated in MATLAB to highlight metabolic profile differences among the five stages.

Moreover, we performed PLS-DA on the SRV data to check grouping trends. The supervised approach used class membership information to attempt at the maximum segregation among the groups of the rats. Furthermore, RPT was utilized to determine the reliability of the sample classification [65], and assess the risk that the current PLS-DA was spurious [66]. This validation is usually performed by comparing the goodness of fit (R^2^ and Q^2^) of the original model with that of several models based on data where the order of the Y-observations is randomly permuted, while the X-matrix is kept intact. If the original model is valid, all the Q^2^-values to the left are lower than the original points to the right, and the regression line of the Q^2^-points intersects the vertical axis below zero [67]. Both PLS-DA and the corresponding RPT were conducted by using SIMCA-P+ 12.0.

OPLS-DA is a derivative PLS-DA removing uncorrelated variables in the within-class with the orthogonal filter [16, 64]. Most of the variables related to the class belonging are described on the first predictive principal component in the OPLS-DA model. Then, the linear classifiers were created on the basis of the PLS-DA models and OPLS-DA models in MATLAB (www.mathworks.com/help/stats/discriminant-analysis.html). The linear classifiers were used to verify the accuracy of classification [68]. From the OPLS-DA loading plots, we identified differential metabolites significantly responsible for the class separation. Two criterions were used for the identification: one is the variable importance value (VIP) in the projection [64], another is the correlation coefficients (r) of the variables relative to the first predictive component (tp1) in the OPLS-DA model [69]. The table of critical values of correlation coefficient (r) was referred based on the degrees of freedom (df). The df values were determined as n1+n2-2 with n1 and n2 as the respective number of samples of the two groups in the OPLS-DA model. The loading plots of the OPLS-DA model with the two criterions were reconstituted in MATLAB. In the reconstituted loading plots, the red color indicates the peak with VIP > 1 and |r| > the critical value of P = 0.01; orange indicates the peak with VIP > 1 and |r| between the critical values of P = 0.05 and P = 0.01; blue indicates the peak with VIP < 1 or |r| < the critical value of P = 0.05.

### R-STOCSY for assigning metabolite resonances

The R-STOCSY analysis was developed by a combination of SRV with the STOCSY analysis [13]. STOCSY can not only provide the hallmark information of spin correlations contained in a classical TOCSY spectrum (intra-metabolite corrections in spin systems), but also offer significant information of metabolic correlations (inter-metabolite corrections in metabolic systems). R-STOCSY represents the autocorrelation matrix of a spectral data set as a 2D pseudo-spectrum [9]. The autocorrelation matrix between the SRV clusters identified from the NMR data set was calculated in MATLAB, according to [9]

$$C=\frac{1}{NS-1}X^{'}X$$

Where, NS is the number of spectra in the data set, X represents the autoscaled SRV cluster matrix of NS spectra × NV SRV clusters, C is the autocorrelation matrix (NV× NV).

Compared to STOCSY, R-STOCSY removes random correlations from the noise that otherwise deteriorate the quality of the STOCSY spectrum [9]. Therefore, R-STOCSY can be used to identify only meaningful correlations between metabolic signals, and significantly enhances the biological interpretation of the STOCSY spectrum. In addition, R-STOCSY is validated through measuring the distances between correlated metabolites within the whole metabolic network, which shows that the average shortest path length is significantly shorter for the detected correlations compared with metabolite couples randomly selected from within the entire KEGG metabolic network [9].

### OR-STOCSY for identifying inter-metabolite correlations

As one of the preprocessing methods, the orthogonal filter is extensively used to remove undesirable systemic variation within the data set [16, 65]. The OR-STOCSY analysis was developed by associating the orthogonal filter with R-STOCSY [15]. As a supervised approach, OR-STOCSY yields pairwise inter-metabolite correlations related to perturbations of biological interest, even if they make a minor contribution to the global variance of a complex data compared to other (possible confounding) effects under study [15].

### Reconstructing perturbed metabolic networks

The metabolism is the set of life-sustaining chemical transformations within the cells of living organisms which include thousands of reactions between metabolites and enzymes. As a comprehensive knowledge repository, the Kyoto Encyclopedia of Genes and Genomes (KEGG, www.genome.jp/kegg/) has been extensively utilized as one of the main data resources to reconstruct metabolic networks [70]. The metabolic network can be viewed both as a network of proteins (metabolic enzymes) and as a network of chemical compounds (metabolites). In the KEGG database, the information of one metabolic pathway is separately stored into one separate KGML file (KEGG Markup Language, www.genome.jp/kegg/xml/) [71]. The information contains metabolites, enzymes and metabolite-enzyme relations, and involves many different species (human, rats, mice, etc.). To more efficiently explore a global metabolic network for rats, we integrated all rat-related information contained in KGML files into one bio-graph object file with our homebuilt scripts executed in MATLAB. The bio-graph object file was developed specially for bioinformatics analysis, using nodes to represent metabolites and enzymes, edges to denote metabolite-enzyme relations. The bio-graph object file contains 70 metabolic pathways, including 2501 nodes (1183 metabolites, 1318 enzymes) and 2862 relations. This novel network analysis tool is provided herein as Supplementary Materials and Methods, including the scripts with instructions and the edited KEGG PATHWAY database.

From the OR-STOCSY analyses of GS, LGD, HGD, GC rats vs. CON rats, we identified significant inter-metabolite correlations linked to the four pathological stages with a correlation threshold of 0.9. Through calculating the shortest path lengths among the corrected metabolites [17], we extracted significant metabolites, enzymes and metabolite-enzyme relations from the bio-graph object file to reconstruct perturbed metabolic networks associated with the four pathological stages.

### Identifying significantly altered metabolic pathways

A perturbed metabolic network is consist of several metabolic pathways linked by metabolites, enzymes and metabolites-enzyme relations. Expectedly, metabolic alterations occurring in important nodes of the metabolic network would potentially trigger significant impacts on the pathway than those occurring in marginal or relatively isolated nodes [72]. The architecture of metabolic pathways represents the knowledge about the complicated relationships among molecules within a global metabolic network. The pathway topology analysis has been used to identify metabolic pathways which are significantly altered under conditions of study [18]. The relative-betweenness centrality arithmetic is usually used to measure the number of shortest paths going through the node. We conducted the pathway topology analysis in MetaboAnalyst 3.0 (www.metaboanalyst.ca/) [73] to calculate pathway impact values based on the metabolites which were used to reconstruct the metabolic network. Thereafter, we identified the significantly altered metabolic pathways according to the calculated pathway impact values with a threshold of 0.3 [18].

## SUPPLEMENTARY DATA FIGURES AND TABLES




