# Supplementary material for: Metabolomic analysis reveals altered metabolic pathways in a rat model of gastric carcinogenesis
Source: Oncotarget. 2016 Aug 4;7(37):60053–73. doi: 10.18632/oncotarget.11049 (PMC5312368; doi:10.18632/oncotarget.11049)
Supplement: Supplementary file 1 [file oncotarget-07-60053-s001.docx]

**Supplementary Table 1.** Intra-metabolite correlations among all clusters derived from the pseudo-2D R-STOCSY spectrum (Figure S2).

| No.* | Correlated SRV cluster IDs^*^ | Chemical shift area (ppm) | Intra-metabolite correlations |
| --- | --- | --- | --- |
| 1 | 139-138 | 1.072-1.092 | isobutyrate |
| 2 | 136-135 | 1.134-1.199 | ethanol |
| 3 | 128-127 | 1.454-1.483 | alanine |
| 4 | 124-123 | 1.530-1.611 | citrulline |
| 5 | 122-121 | 1.623-1.679 | lysine |
| 6 | 119-118 | 1.710-1.769 | lysine |
| 7 | 116-114 | 1.840-1.971 | ornithine |
| 8 | 113-112 | 1.992-2.019 | proline |
| 9 | 111-110 | 2.060-2.085 | α-acid glycoprotein |
| 10 | 111-110,23-22 | 2.060-2.085, 6.987-6.999 | α-acid glycoprotein |
| 11 | 106-105 | 2.128-2.177 | glutamine |
| 12 | 103-100 | 2.189-2.256 | unassigned |
| 13 | 103-100,98-95 | 2.189-2.256, 2.294-2.454 | 3-hydroxybutyrate |
| 14 | 103-101,92-91 | 2.189-2.235, 2.534-2.650 | citrate |
| 15 | 103-101,89-88 | 2.189-2.235, 2.725-2.855 | PUFA |
| 16 | 103-101,80 | 2.189-2.235, 3.118-3.128 | histidine |
| 17 | 99,35-34 | 2.275-2.284, 4.345-4.477 | unassigned |
| 18 | 98-95 | 2.294-2.454 | glutamine |
| 19 | 92-91 | 2.534-2.638 | citrate |
| 20 | 89-88 | 2.725-2.855 | PUFA |
| 21 | 87-86 | 2.872-2.999 | asparagine |
| 22 | 72-71 | 3.282-3.309 | unassigned |
| 23 | 69-68 | 3.328-3.358 | proline |
| 24 | 69-68,40 | 3.328-3.358, 4.182-4.191 | proline, |
| 25 | 38,72-71 | 4.232-4.242, 3.282-3.309 | unassigned |
| 26 | 66-65 | 3.377-3.447 | taurine |
| 27 | 65,31 | 3.397-3.447, 4.675-4.685 | taurine, |
| 28 | 65,21 | 3.397-3.447, 7.024-7.035 | taurine, |
| 29 | 62-61 | 3.492-3.612 | glucose |
| 30 | 64,62-61 | 3.448-3.458, 3.492-3.612 | glucose |
| 31 | 60-59 | 3.634-3.708 | glucose |
| 32 | 58-56 | 3.709-3.772 | glucose |
| 33 | 31,54 | 4.675-4.685, 3.813-3.823 | glucose |
| 34 | 51-50 | 3.910-3.937 | glucose |
| 35 | 51-50,30 | 3.910-3.937, 4.697-5.602 | glucose |
| 36 | 51-50,25-24 | 3.910-3.937, 6.963-6.986 | unassigned |
| 37 | 49-48 | 3.948-3.970 | serine |
| 38 | 47-45 | 3.984-4.059 | serine |
| 39 | 44-43 | 4.082-4.139 | lactate |
| 40 | 42-41 | 4.140-4.173 | 3-hydroxybutyrate |
| 41 | 42,21 | 4.140-4.151, 7.024-7.035 | 3-hydroxybutyrate, |
| 42 | 37-33 | 4.289-4.653 | GPC |
| 43 | 25,34-33 | 6.963-6.976, 4.082-4.139 | glucose |
| 44 | 24,32 | 6.977-6.986, 4.654-4.674 | glucose |
| 45 | 30-29 | 4.697-5.716 | unassigned |
| 46 | 25-24,30-29 | 6.963-6.986, 4.697-5.716 | unassigned |
| 47 | 28-27 | 6.309-6.899 | tyrosine |
| 48 | 25-24 | 6.963-6.986 | unassigned |
| 49 | 23-22 | 6.987-7.009 | 3-methylhistidine |
| 50 | 20-19 | 7.055-7.190 | histidine |
| 51 | 21,6 | 7.024-7.035, 7.776-7.791 | histidine |
| 52 | 17-16 | 7.210-7.326 | phenylalanine |
| 53 | 14-11 | 7.346-7.414 | phenylalanine |
| 54 | 9-8 | 7.429-7.753 | phenylalanine |
| 55 | 4-2 | 8.452-8.595 | formate |

No.^*^ is the number shown in the R-STOCSY spectrum; SRV cluster IDs^*^ are the numbers of SRV clusters data derived from the NMR spectra.

**Supplementary Table 7.** Inter-metabolic correlations identified from the OR-STOCSY analysis of GS rats vs. CON rats (Figure 6A).

| No.^*^ | Correlated SRV cluster IDs^*^ | Chemical shift area (ppm) | Inter-metabolite correlations |
| --- | --- | --- | --- |
| 1 | 148,139-138 | 0.828-0.919, 1.072-1.092 | lipid, isobutyrate |
| 2 | 148,131 | 0.828-0.919, 1.331-1.385 | lipid, lactate |
| 3 | 139-138,144-143 | 1.072-1.092, 0.968-0.994 | valine, leucine, isobutyrate |
| 4 | 136,128-127 | 1.134-1.163,1.454-1.469 | ethanol, alanine |
| 5 | 119-118,115 | 1.710-1.769, 1.914-1.924 | lysine, acetate |
| 6 | 122-121,102-101 | 1.623-1.670, 2.207-2.235 | lysine, valine |
| 7 | 121,96 | 2.207-2.217, 2.394-2.429 | lysine,3-hydroxybutyrate |
| 8 | 122-121,92-91 | 1.623-1.670, 2.534-2.650 | lysine, citrate |
| 9 | 122,89 | 1.623-1.637, 2.725-2.774 | lysine, PUFA |
| 10 | 122-121,80 | 1.623-1.670, 3.118-3.128 | lysine, histidine |
| 11 | 102-100,96-95 | 2.207-2.256, 2.394-2.454 | valine, glutamine |
| 12 | 103-100,92-89 | 2.189-2.256, 2.534-2.725 | valine, citrate |
| 13 | 103-101,80 | 2.189-2.235, 3.118-3.128 | valine, histidine |
| 14 | 105,69 | 2.139-2.177, 3.328-3.337 | glutamine, proline |
| 15 | 103-101,65 | 2.189-2.235, 3.397-3.447 | valine, glucose |
| 16 | 103-101,23-22 | 2.189-2.235, 6.987-7.009 | valine,3-methylhistidine |
| 17 | 98,6 | 2.294-2.305, 7.776-7.791 | 3-hydroxybutyrate,histidine |
| 18 | 92-89,96-95 | 2.534-2.725, 2.394-2.454 | citrate, PUFA, glutamine |
| 19 | 80,96-95 | 3.118-3.128, 2.394-2.454 | histidine, glutamine |
| 20 | 80,92-91 | 3.118-3.128, 2.534-2.650 | histidine, citrate |
| 21 | 80,89 | 3.118-3.128, 2.725-2.774 | histidine, PUFA |
| 22 | 66-65,96-95 | 3.377-3.447, 2.394-2.454 | glucose, glutamine |
| 23 | 65,92-91 | 3.397-3.447, 2.534-2.650 | glucose, citrate |
| 24 | 66-65,80 | 3.377-3.447, 3.118-3.128 | glucose, histidine |
| 25 | 21,98 | 7.024-7.035, 2.294-2.305 | 1-methylhistidine,  3-hydroxybutyrate |
| 26 | 23-22,96-95 | 6.987-7.009, 2.394-2.454 | 3-methylhistidine,glutamine |
| 27 | 23-22,92-91 | 6.987-7.009, 2.534-2.650 | 3-methylhistidine,citrate |
| 28 | 23-22,89 | 6.987-7.009, 2.725-2.774 | 3-methylhistidine, PUFA |
| 29 | 23-22,80 | 6.987-7.009, 3.118-3.128 | 3-methylhistidine,histidine |
| 30 | 23-22,66-65 | 6.987-7.009, 3.377-3.447 | 3-methylhistidine,glucose |
| 31 | 17-16,4 | 7.210-7.326, 8.454-8.466 | phenylalanine, formate |

No.^*^ is the number shown in the pseudo-2D OR-STOCSY spectrum; SRV cluster IDs^*^ are the numbers of SRV clusters data derived from the NMR spectra.

**Supplementary Table 8.** Inter-metabolite correlations identified from the OR-STOCSY analysis of LGD rats vs. CON rats (Figure 6B).

| No.^*^ | Correlated SRV cluster IDs^*^ | Chemical shift area (ppm) | Inter-metabolite correlations |
| --- | --- | --- | --- |
| 1 | 145,141 | 0.957-0.967, 1.032-1.044 | leucine, valine |
| 2 | 139-138,144-143 | 1.072-1.092, 0.968-0.994 | isobutyrate, leucine, valine |
| 3 | 136-135,128-127 | 1.134-1.199, 1.454-1.483 | 3-hydroxybutyrate,alanine |
| 4 | 119,115 | 1.710-1.737, 1.914-1.924 | lysine, acetate |
| 5 | 102,96 | 2.207-2.217, 2.394-2.429 | valine,3-hydroxybutyrate |
| 6 | 104,70 | 2.178-2.188, 3.316-3.327 | glutamine, proline |
| 7 | 111,66-65 | 2.020-2.059, 3.377-3.447 | α-acid glycoprotein, glucose |
| 8 | 111-110,23-22 | 2.020-2.085, 6.987-7.009 | α-acid glycoprotein, 3-methylhistidine |
| 9 | 111-110,21 | 2.020-2.085, 7.024-7.035 | α-acid glycoprotein, 1-methylhistidine |
| 10 | 111-110,6 | 2.020-2.085, 7.776-7.791 | α-acid glycoprotein, histidine |
| 11 | 66-65,23-22 | 3.377-3.447, 6.987-7.009 | glucose, 3-methylhistidine |
| 12 | 66-65,6 | 6.987-7.009, 7.776-7.791 | glucose, histidine |
| 13 | 21,66-65 | 7.024-7.035, 6.987-7.009 | 1-methylhistidine, glucose |
| 14 | 22,54 | 6.999-7.009, 3.813-3.823 | 3-methylhistidine, glucose |
| 15 | 5,51 | 7.835-7.844, 3.910-3.922 | xanthine, glucose |
| 16 | 22,31 | 6.999-7.009, 4.675-4.685 | 3-methylhistidine, glucose |
| 17 | 30,5 | 4.697-5.602, 7.835-7.844 | glucose, xanthine |
| 18 | 6,23-22 | 7.776-7.791, 6.987-7.009 | histidine,3-methylhistidine |
| 19 | 17-16,4 | 7.210-7.326, 8.454-8.466 | phenylalanine, formate |
| 20 | 6,21 | 7.776-7.791, 7.024-7.035 | histidine,1-methylhistidine |

No.^*^ is the number shown in the pseudo-2D OR-STOCSY spectrum; SRV cluster IDs^*^ are the numbers of SRV clusters data derived from the NMR spectra.

**Supplementary Table 9.** Inter-metabolite correlations identified from the OR-STOCSY analysis of HGD rats vs. CON rats (Figure 6C).

| No.^*^ | Correlated SRV cluster IDs^*^ | Chemical shift area (ppm) | Inter-metabolite  correlations |
| --- | --- | --- | --- |
| 1 | 145,141 | 0.957-0.96, 1.032-1.044 | leucine, valine |
| 2 | 139-138,144-143 | 1.072-1.092, 0.968-0.994 | isobutyrate, leucine, valine |
| 3 | 136-135,128-127 | 1.134-1.199, 1.454-1.483 | 3-hydroxybutyrate, alanine |
| 4 | 122-121,102-101 | 1.623-1.679, 2.207-2.235 | lysine, valine |
| 5 | 122-121,92-91 | 1.623-1.679, 2.534-2.650 | lysine, citrate |
| 6 | 122-121,89 | 1.623-1.679, 2.725-2.774 | lysine, PUFA |
| 7 | 122-121,66-65 | 1.623-1.679, 3.377-3.447 | lysine, glucose |
| 8 | 111-110,23-22 | 2.020-2.085, 6.987-7.009 | α-acid glycoprotein, 3-methylhistidine |
| 9 | 111,6 | 2.020-2.059, 7.776-7.791 | α-acid glycoprotein, histidine |
| 10 | 111-110,102-101 | 2.020-2.085, 2.207-2.235 | α-acid glycoprotein, valine |
| 11 | 111,96 | 2.020-2.059, 2.394-2.429 | α-acid glycoprotein, 3-hydroxybutyrate,  succinate |
| 12 | 111,92-91 | 2.020-2.059, 2.534-2.650 | α-acid glycoprotein, citrate |
| 13 | 111-110,66-65 | 2.020-2.085, 3.377-3.447 | α-acid glycoprotein, glucose |
| 14 | 102-101,96-95 | 2.207-2.235, 2.395-2.454 | valine, glutamine |
| 15 | 102-101,92-91 | 2.207-2.235, 2.534-2.650 | valine, citrate |
| 16 | 104,70 | 2.178-2.188, 3.316-3.327 | glutamine, proline |
| 17 | 96,92-91 | 2.394-2.429, 2.534-2.650 | succinate, citrate |
| 18 | 89,102-101 | 2.725-2.855, 2.207-2.235 | PUFA, valine |
| 19 | 89,96 | 2.725-2.774, 2.394-2.429 | PUFA, 3-hydroxybutyrate,  succinate |
| 20 | 66-65,102-101 | 3.377-3.447, 2.207-2.235 | glucose, valine |
| 21 | 65,96 | 3.397-3.447, 2.394-2.429 | glucose, 3-hydroxybutyrate,  succinate |
| 22 | 66-65,92-91 | 3.377-3.447, 2.534-2.650 | glucose, citrate |
| 23 | 66-65,89 | 3.377-3.447, 2.725-2.774 | glucose, PUFA |
| 24 | 23-22,102-101 | 6.987-7.009, 2.207-2.235 | 3-methylhistidine,valine |
| 25 | 23-22,96 | 6.987-7.009, 2.394-2.429 | 3-methylhistidine,  3-hydroxybutyrate,succinate |
| 26 | 23-22,92-91 | 6.987-7.009, 2.534-2.650 | 3-methylhistidine,citrate |
| 27 | 23-22,66-65 | 6.987-7.009, 3.377-3.447 | 3-methylhistidine,glucose |
| 28 | 21,102-101 | 7.024-7.035, 2.207-2.235 | 1-methylhistidine,valine |
| 29 | 21,92-91 | 7.024-7.035, 2.534-2.650 | 1-methylhistidine,citrate |
| 30 | 21,89 | 7.024-7.035, 2.725-2.774 | 1-methylhistidine,PUFA |
| 31 | 6,102-101 | 7.776-7.791, 2.207-2.235 | histidine, valine |
| 32 | 6,96 | 7.776-7.791, 2.394-2.429 | histidine,3-hydroxybutyrate,  succinate |
| 33 | 6,92-91 | 7.776-7.791, 2.534-2.650 | histidine, citrate |
| 34 | 6,89 | 7.776-7.791, 2.725-2.774 | histidine, PUFA |
| 35 | 6,66-65 | 7.776-7.791, 3.377-3.447 | histidine, glucose |
| 36 | 23,6 | 6.987-6.996, 7.776-7.791 | 3-methylhistidine,histidine |
| 39 | 5,30 | 7.835-7.844, 4.697-5.602 | xanthine, glucose |
| 38 | 6,21 | 7.776-7.791, 7.024-7.035 | histidine,1-methylhistidine |
| 39 | 5-4,17 | 7.835-8.466, 7.210-7.220 | formate, phenylalanine |

No.^*^ is the number shown in the pseudo-2D OR-STOCSY spectrum; SRV cluster IDs^*^ are the numbers of SRV clusters data derived from the NMR spectra.

**Supplementary Table 10.** Inter-metabolite correlations identified from the OR-STOCSY analysis of GC rats vs. CON rats (Figure 6D).

| No.^*^ | Correlated SRV cluster IDs^*^ | Chemical shift area (ppm) | Inter-metabolite correlations |
| --- | --- | --- | --- |
| 1 | 146,150 | 0.945-0.956, 0.703-0.750 | leucine, lipid |
| 2 | 144-143,139-138 | 0.968-0.994, 1.072-1.092 | isobutyrate, leucine, valine |
| 3 | 144,135 | 0.968-0.977, 1.187-1.199 | leucine, 3-hydroxybutyrate |
| 4 | 139,135 | 1.072-1.082, 1.187-1.199 | isobutyrate, 3-hydroxybutyrate |
| 5 | 130,139-138 | 1.394-1.404,1.072-1.092 | isoleucine, isobutyrate |
| 6 | 136-135,127 | 1.134-1.199, 1.469-1.483 | ethanol, alanine |
| 7 | 130,119 | 1.394-1.404, 1.710-1.737 | isoleucine, lysine |
| 8 | 130,110 | 1.394-1.404, 2.060-2.085 | isoleucine, glutamate |
| 9 | 139-138,69 | 1.072-1.092, 3.328-3.337 | isobutyrate, proline |
| 10 | 139-138,42 | 1.072-1.092, 4.140-4.151 | isobutyrate, 3-hydroxybutyrate |
| 11 | 139-138,36-35 | 1.072-1.092, 4.334-4.417 | isobutyrate, GPC |
| 12 | 122-121,102-101 | 1.623-1.679, 2.207-2.217 | lysine, valine |
| 13 | 122-121,96 | 1.623-1.679, 2.394-2.429 | lysine, 3-hydroxybutyrate |
| 14 | 122-121,92-91 | 1.623-1.679, 2.534-2.650 | lysine, citrate |
| 15 | 122-121,89 | 1.623-1.679, 2.725-2.774 | lysine, PUFA |
| 16 | 130,44-43 | 1.394-1.404, 4.082-4.139 | isoleucine, lactate |
| 17 | 130,23-22 | 1.394-1.404, 6.987-7.009 | isoleucine, 3-methylhistidine |
| 18 | 110,99 | 2.060-2.085, 2.275-2.284 | PUFA, valine |
| 19 | 102-101,96-95 | 2.207-2.235, 2.394-2.454 | valine, glutamine |
| 20 | 103-101,92-91 | 2.189-2.235, 2.534-2.650 | valine, citrate |
| 21 | 102,89 | 2.207-2.217, 2.725-2.774 | valine, PUFA |
| 22 | 103,80 | 2.189-2.199, 3.118-3.128 | valine, histidine |
| 23 | 104,70 | 2.178-2.188, 3.316-3.327 | glutamine, proline |
| 24 | 111,65 | 2.020-2.059, 3.397-3.447 | α-acid glycoprotein, glucose |
| 25 | 99,44-43 | 2.275-2.284, 4.082-4.139 | valine, lactate |
| 26 | 111-110,23-22 | 2.020-2.085, 6.987-7.009 | α-acid glycoprotein, 3-methylhistidine |
| 27 | 111-110,21 | 2.020-2.085, 7.024-7.035 | α-acid glycoprotein, 1-methylhistidine |
| 28 | 111,6 | 2.020-2.059, 7.776-7.791 | α-acid glycoprotein, histidine |
| 29 | 96-95,92-91 | 2.394-2.454, 2.534-2.650 | 3-hydroxybutyrate,citrate |
| 30 | 89,96 | 2.725-2.774, 2.394-2.429 | PUFA,3-hydroxybutyrate |
| 31 | 80,101 | 3.118-3.128, 2.225-2.235 | histidine, valine |
| 32 | 80,92-91 | 3.118-3.128, 2.534-2.650 | histidine, citrate |
| 33 | 80,89 | 3.118-3.128, 2.725-2.774 | histidine, PUFA |
| 34 | 69,99 | 3.328-3.337, 2.275-2.284 | valine, proline |
| 35 | 57-56,102-101 | 3.750-3.761, 2.207-2.235 | alanine, valine |
| 36 | 56,96 | 3.762-3.772, 2.394-2.429 | alanine, succinate, 3-hydroxybutyrate |
| 37 | 56,92-91 | 3.762-3.772, 2.534-2.650 | alanine, citrate |
| 38 | 96,89 | 2.394-2.429, 2.725-2.774 | 3-hydroxyvutyrate, PUFA |
| 39 | 56,80 | 3.762-3.772, 3.118-3.128 | alanine, histidine |
| 40 | 36-34,99 | 4.334-4.477, 2.275-2.284 | GPC, proline |
| 41 | 42-41,80 | 4.140-4.173, 3.118-3.128 | 3-hydroxybutyrate, histidine |
| 42 | 69-68,44-41 | 3.328-3.358, 4.082-4.172 | proline, lactate, 3-hydroxybutyrate |
| 43 | 69-68,37-34 | 3.328-3.358, 4.289-4.477 | proline, GPC |
| 44 | 65,22 | 3.397-3.447, 6.999-7.009 | glucose,  3-methylhistidine |
| 45 | 20,58-57 | 7.055-7.066,3.709- 3.772 | histidine, glucose |
| 46 | 44-43,49-48 | 4.082-4.139, 3.948-3.970 | lactate, serine |
| 47 | 44-41,36-34 | 4.082-4.139, 4.334-4.477 | lactate,  3-hydroxybutyrate, GPC |
| 48 | 42,23-22 | 4.140-4.151, 6.987-7.009 | 3-hydroxybutyrate,  3-methylhihstidine |
| 49 | 26-25,44-43 | 6.900-6.976, 4.082-4.139 | tyrosine, lactate |
| 50 | 6,21 | 6.776-7.791, 7.024-7.035 | histidine,  1-methylhistidine |
| 51 | 23-22,6 | 6.987-7.009, 7.776-7.791 | 3-methylhistidine, histidine |
| 52 | 17,4-3 | 7.210-7.220, 8.454-8.595 | phenylalanine, formate |

No.^*^ is the number shown in the pseudo-2D OR-STOCSY spectrum; SRV cluster IDs^*^ are the numbers of SRV clusters data derived from the NMR spectra.
