# Supplementary material for: Metabolomic analysis reveals altered metabolic pathways in a rat model of gastric carcinogenesis
Source: Oncotarget. 2016 Aug 4;7(37):60053–73. doi: 10.18632/oncotarget.11049 (PMC5312368; doi:10.18632/oncotarget.11049)
Supplement: Supplementary file 2 [file oncotarget-07-60053-s002.pdf]

Metabolomic analysis reveals altered metabolic pathways in a rat model of gastric carcinogenesis

SUPPLEMENTARY DATA

This RAR file contains 5 scripts of MATLAB function, 1 m.file, which is the demo of these 5 scripts, 2 data and 3 KGML files.

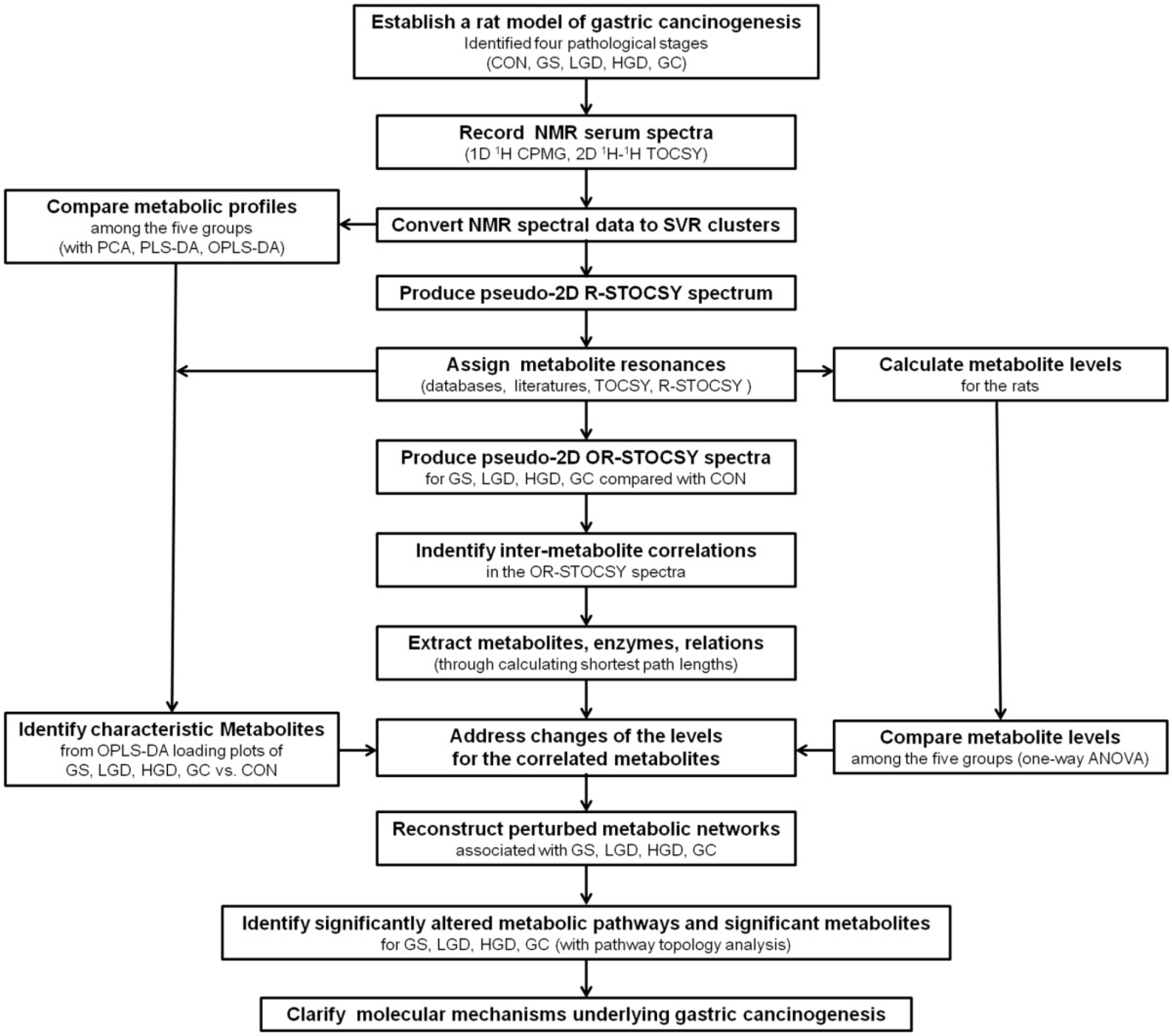

Supplementary Figure S1: Flowchart of the NMR-based metabonomic analysis for revealing the significantly altered metabolic pathways associated with four typical pathological stages of gastric carcinogenesis.

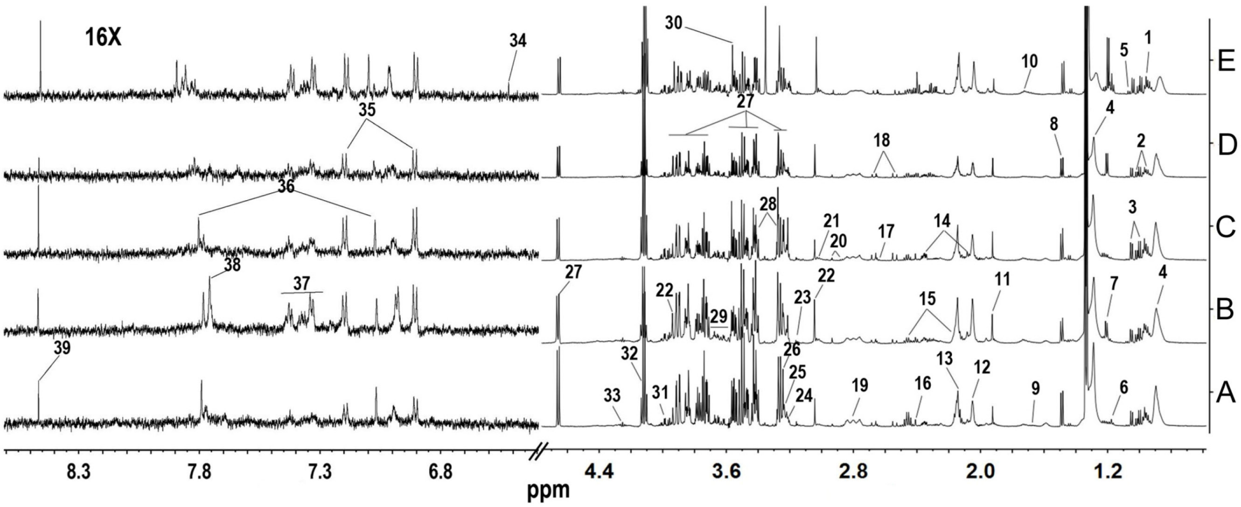

Supplementary Figure S2: Typical 600 MHz 1D ^{1}H CPMG spectra of the sera derived from the CON rats and MODEL rats in four typical pathological stages of gastric carcinogenesis including CON **A.** GS **B.** LGD **C.** HGD **D.** GC **E.** The serial numbers indicate the following metabolites: 1, leucine; 2, isoleucine; 3, valine; 4, lipid; 5, isobutyrate; 6, ethanol; 7, 3-hydrxoybutyrate; 8, alanine; 9, arginine; 10, lysine; 11, acetate; 12, NAc1*; 13; NAc2*; 14, glutamate; 15, glutamine; 16, succinate; 17, aspartate; 18, citrate; 19, PUFA; 20, asparagine; 21, ornithine; 22, creatine; 23, citrulline; 24, choline; 25, phosphorylcholine (PC); 26, GPC; 27, glucose; 28, taurine; 29, glycerol; 30, glycine; 31, serine; 32, lactate; 33, threonine; 34, fumarate; 35, tyrosine; 36, histidine; 37, phenylalanine; 38, xanthine; 39, formate.

* Both NAc1 and NAc2 refer to composite acetyl signals from α-acid glycoprotein.

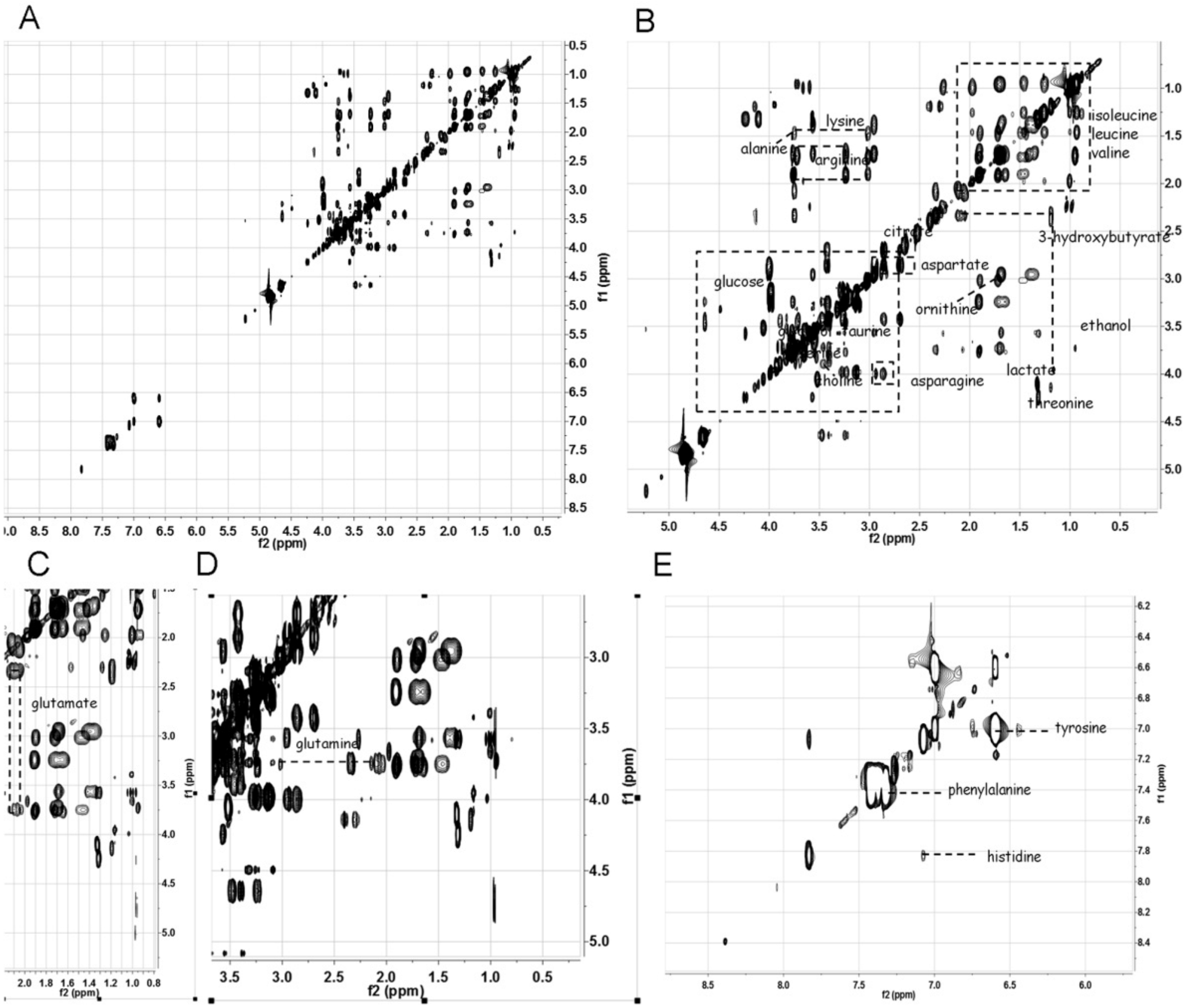

Supplementary Figure S3: Typical 600MHz 2D ^{1}H-^{1}H TOCSY spectrum of a GS rat serum. A. the whole spectrum, **B, C, D, E.** the partial spectrum for verifying the metabolite resonances. The spectrum was recorded using the following parameters: f1= 512 data points (SW ^{1}H: 10 ppm), f2= 2048 data points (SW ^{1}H: 10 ppm), τ_m= 80 ms.

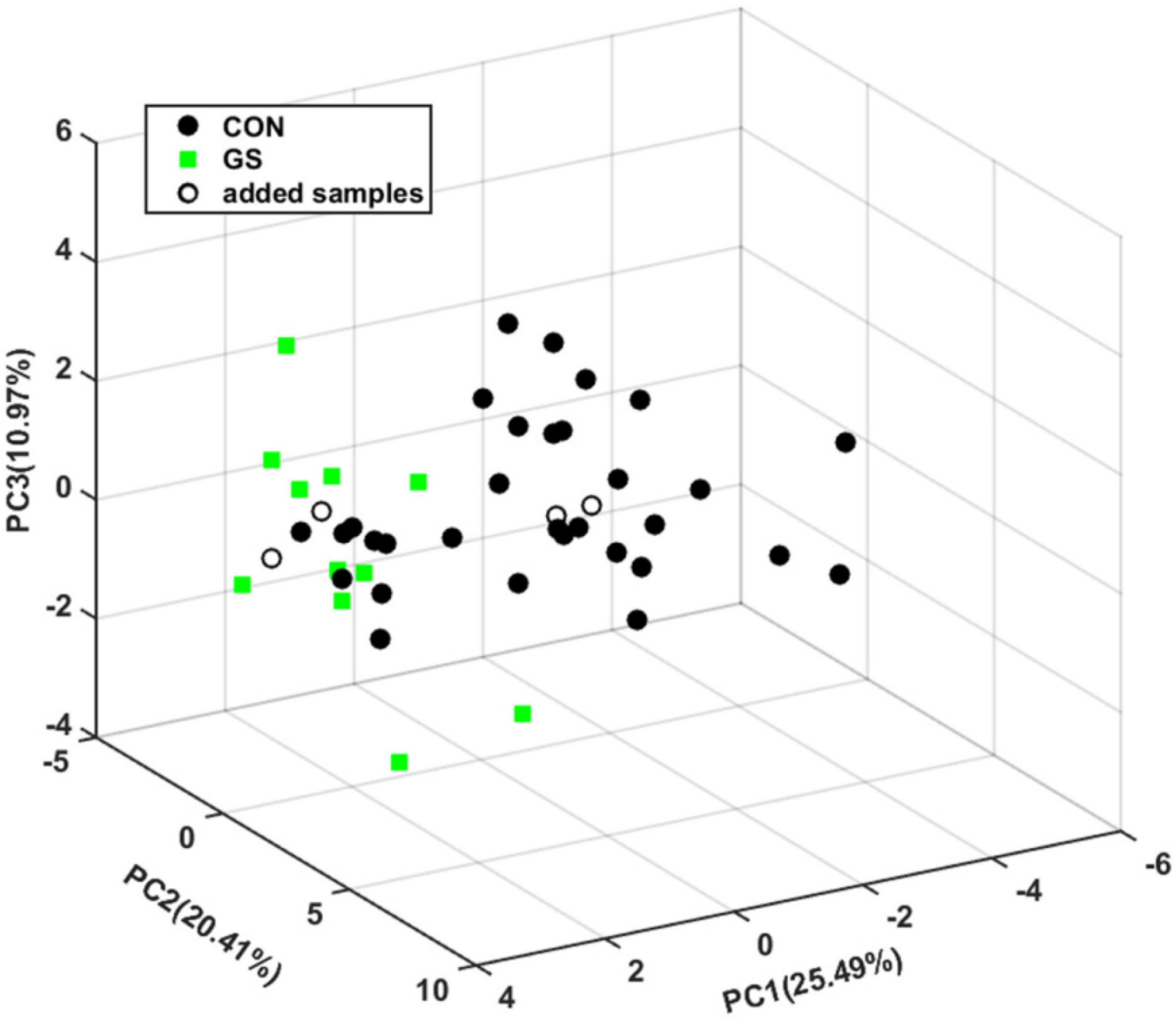

Supplementary Figure S4: The newly-produced PCA scores plot of SRV data from CON rats, GS rats and four extra rats.

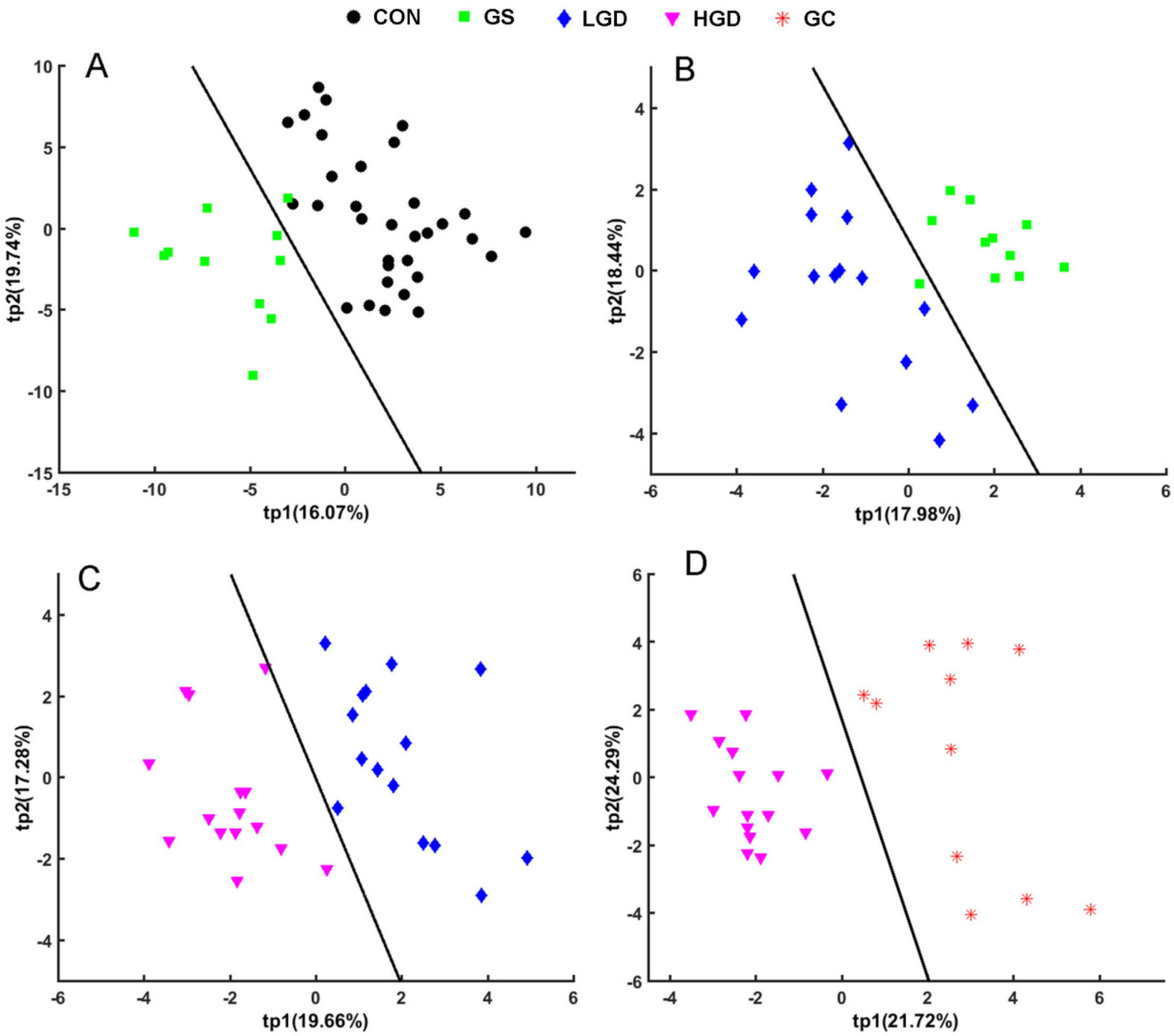

Supplementary Figure S5: PLS-DA scores plots of SRV clusters data derived from 1D ^{1}H CPMG spectra of the sera. The scores plots show clear separation of GS rats from CON rats **A.** LGD rats from GS rats **B.** HGD rats from LGD rats **C.** GC rats from HGD rats **D.**

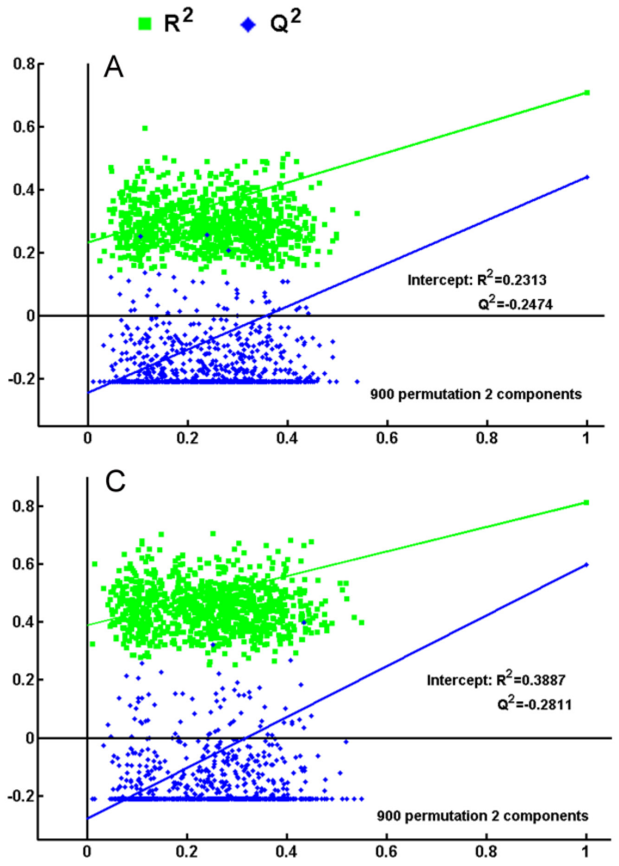

Supplementary Figure S6: The validation plots of the PLS-DA models, generated from the permutation tests that were randomly permuted 900 times with the first two components. The green square is R^2(cum), denoting the explained variance of the model. The blue diamond is Q^2(cum), standing for the predictive ability of the model. **A.** GS rats vs. CON rats; **B.** LGD rats vs. GS rats; **C.** HGD rats vs. LGD rats; **D.** GC rats vs. HGD rats.

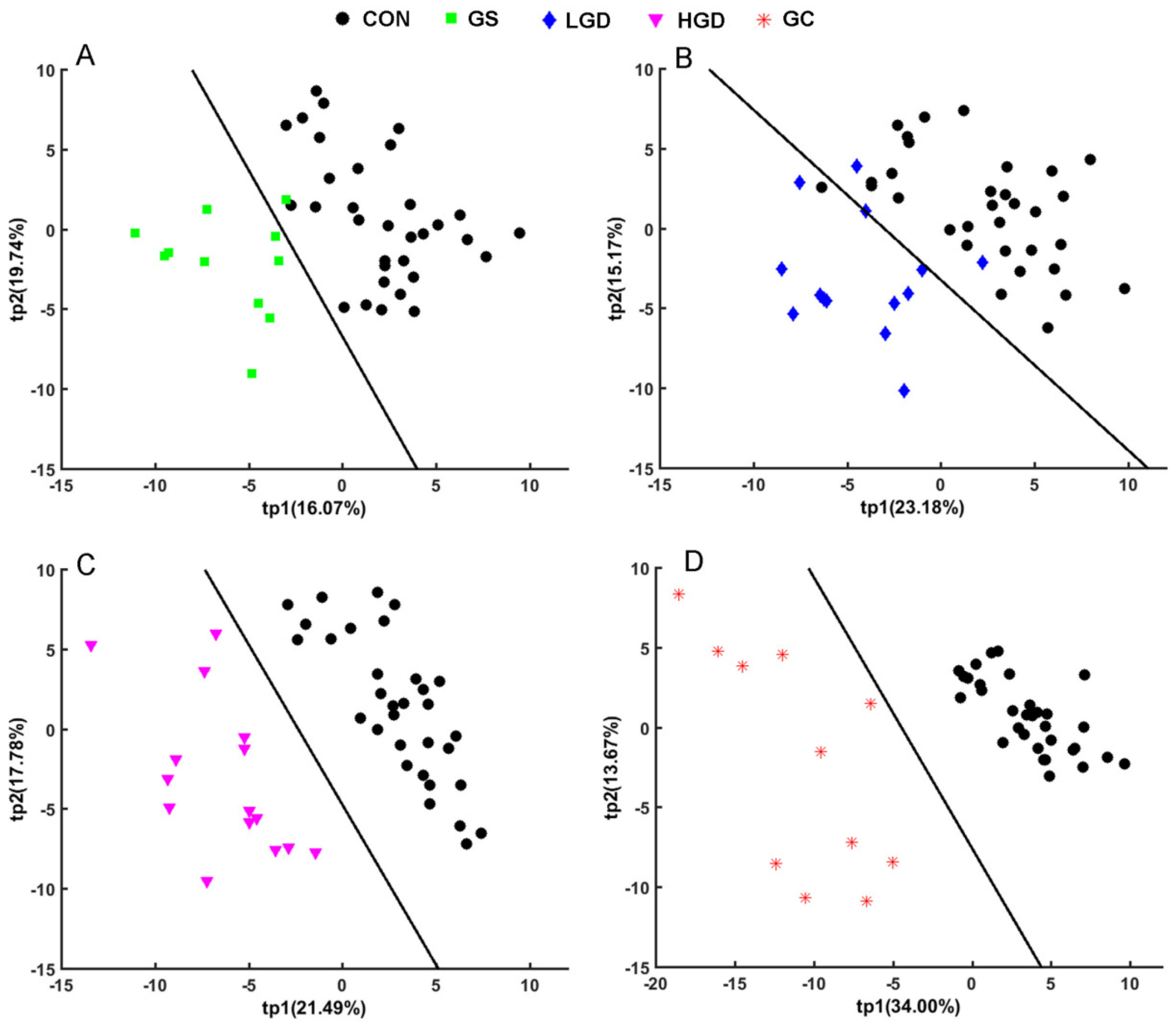

Supplementary Figure S7: PLS-DA scores plots of SRV clusters data derived from 1D ^{1}H CPMG spectra of the sera. The scores plots show clear separation of GS rats **A.** LGD rats **B.** HGD rats **C.** GC rats **D.** from CON rats.

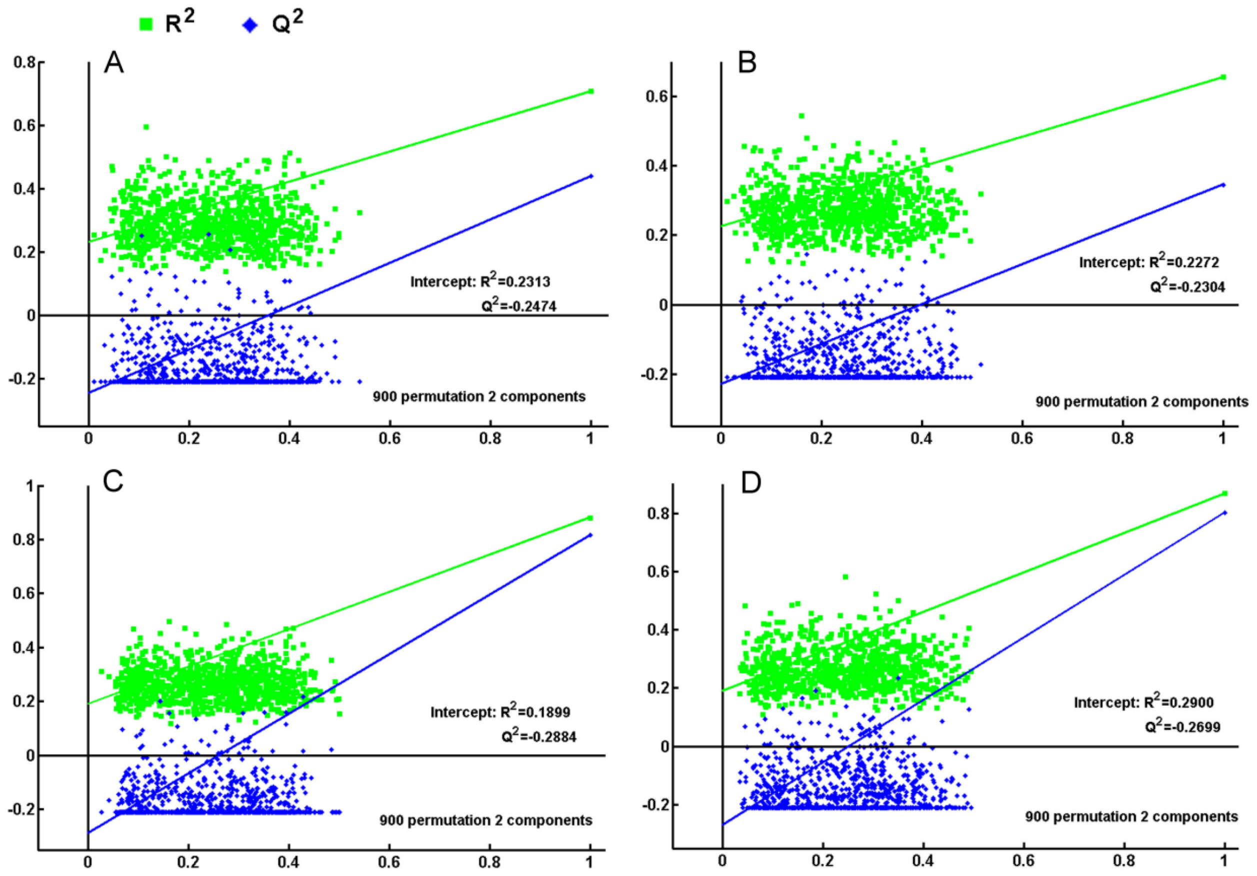

Supplementary Figure S8: The validation plots of the PLS-DA models, generated from the permutation tests that were randomly permuted 900 times with the first two components. The green square is R^2 (cum), denoting the explained variance of the model. The blue diamond is Q^2 (cum), standing for the predictive ability of the model. **A.** GS rats vs. CON rats; **B.** LGD rats vs. CON rats; **C.** HGD rats vs. CON rats; **D.** GC rats vs. CON rats.

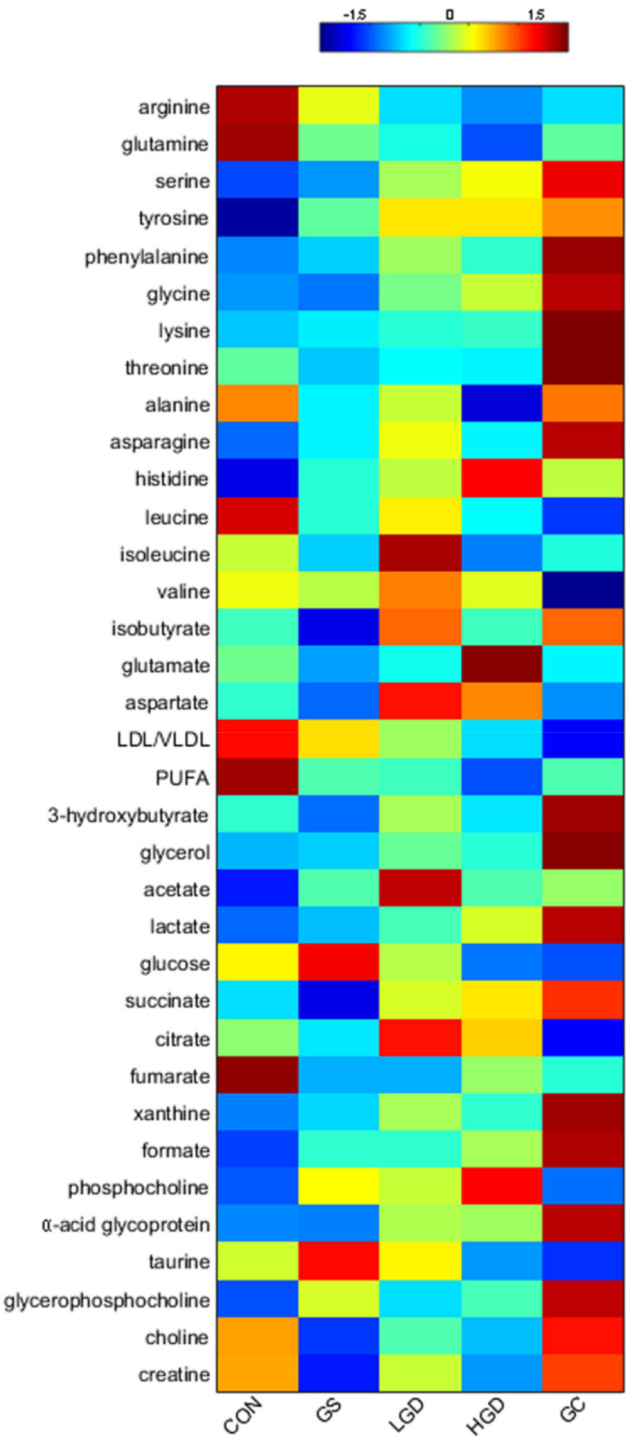

Supplementary Figure S9: The heatmap plot of standardized metabolite levels in the five groups.

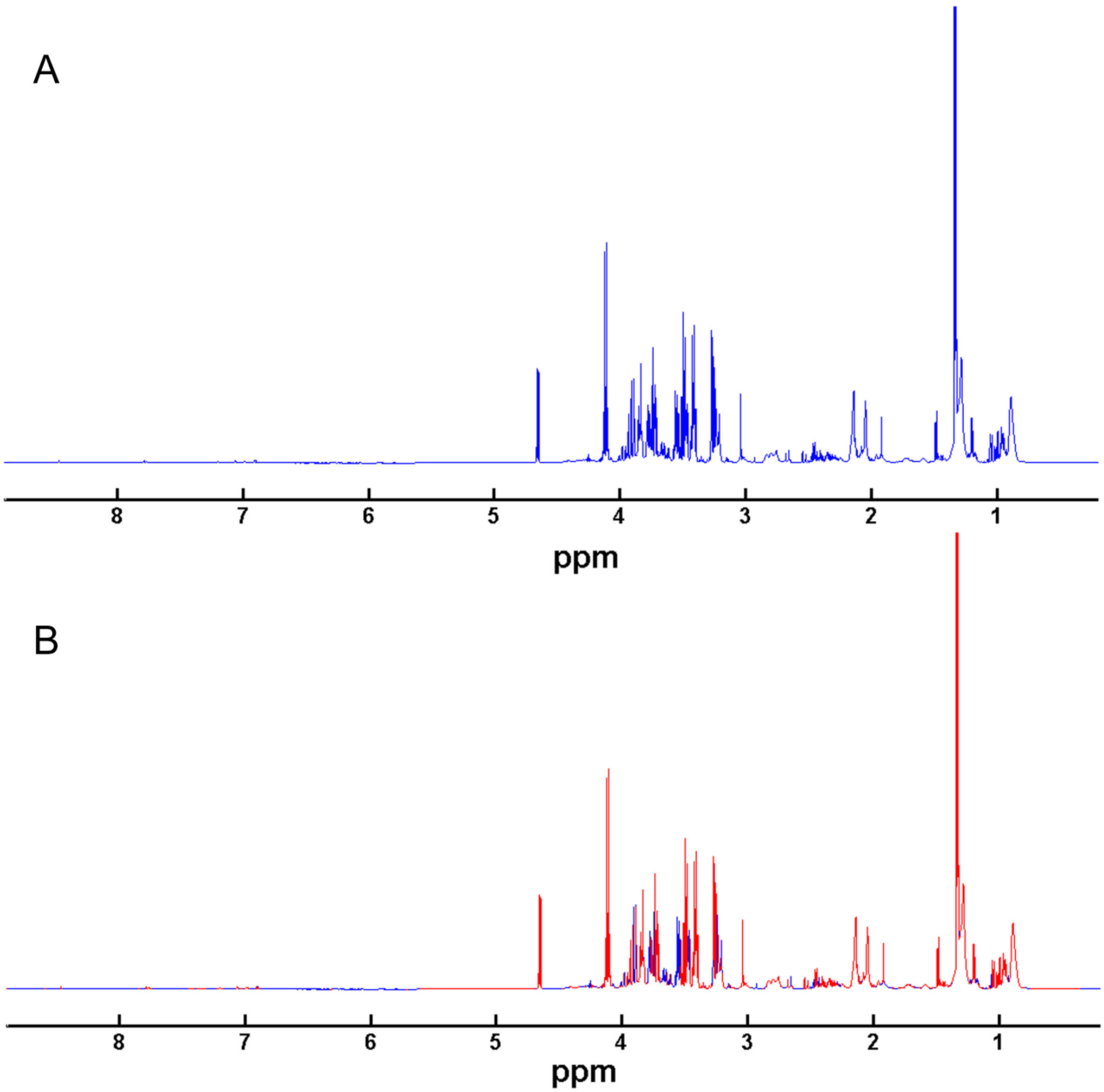

Supplementary Figure S10: Representation of NMR spectra from A. high-resolution bucketing of 1D ^{1}H CPMG spectrum of rat serum, **B.** SRV clusters (red) plotted on the NMR spectrum (blue), illustrating the efficient recovery of NMR signals by the SRV algorithm.

Supplementary Table S1: Intra-metabolite correlations among all clusters derived from the pseudo-2D R-STOCSY spectrum (Supplementary Figure S2)

See Supplementary File 1

Supplementary Table S2: Inter-metabolite correlations among all clusters derived from the pseudo-2D R-STOCSY spectrum (Supplementary Figure S2)

No.*	Correlated SRV cluster IDs*	Chemical shift area (ppm)	Inter-metabolite correlations
56	145-143	0.957-0.994	leucine, valine
57	145-144,141-140	0.957-0.977, 1.032-1.056	leucine, valine
58	144-143,139-138	0.977-0.994, 1.072-1.092	leucine, valine, isobutyrate
59	136-135,128-127	1.134-1.199, 1.454-1.483	ethanol, alanine
60	130,69	1.394-1.404, 3.328-3.337	isoleucine, proline
61	130,44-43	1.394-1.404, 4.082-4.231	isoleuine, lactate
62	130,35	1.394-1.404, 4.345-4.417	isoleuine, GPC
63	122-121,103-101	1.623-1.679, 2.189-2.235	lysine, valine
64	122-121,96-95	1.623-1.679, 2.394-2.454	lysine,3-hydroxybutyrate
65	122-121,92-88	1.623-1.679, 2.524-2.855	lysine, citrate, PUFA
66	122-121,80	1.623-1.679, 3.118-3.128	lysine, histidine
67	115,119-118	1.914-1.924, 1.710-1.769	acetate, lysine
68	44,119-118	4.082-4.100, 1.710-1.769	lactate, lysine
69	113-112,57	1.992-2.019, 3.750-3.761	proline, alanine
70	113-112,20-19	1.992-2.019, 7.055-7.190	proline, histidine
71	104,70	2.178-2.188, 3.316-3.327	glutamine, proline
72	99,49-48	2.275-2.284, 3.948-3.970	valine, serine
73	99,44-43	2.275-2.284, 4.082-4.101	valine, lactate
74	92-91,98	2.534-2.650, 2.294-2.305	citrate, glutamine
75	96-95,92-91	2.394-2.454, 2.524-2.650	carnitine, glutamine
76	89-88,96-95	2.725-2.855, 2.394-2.454	PUFA, carnitine
77	80,96-95	3.118-3.128, 2.394-2.454	carnitine, histidine
78	89-88,92-91	2.725-2.855, 2.534-2.638	PUFA, citrate
79	80,92-91	3.118-3.128, 2.534-2.638	histidine, citrate
80	80,89-88	3.118-3.128, 2.725-2.855	histidine, PUFA
81	69-68,44-43	3.328-3.358, 4.082-4.139	proline, lactate
82	69-68,37-34	3.328-3.358, 4.289-4.477	proline, GPC
83	65,6	3.397-3.447, 7.776-7.791	taurine, histidine
84	20-19,58-56	7.055-7.190, 3.709-3.772	histidine, glucose
85	49-48,44-43	3.948-3.970, 4.082-4.139	serine, lactate
86	44-43,40	4.082-4.139, 4.182-4.191	lactate, threonine
87	36-35,40	4.334-4.345, 4.182-4.191	GPC, threonine
88	44-43,37-34	4.082-4.139, 4.289-4.477	lactate, GPC
89	42,6	4.140-4.151, 7.776-7.791	3-hydroxybutyrate,histidine
90	36,29	4.334-4.344, 5.706-5.716	GPC, uracil
91	25,37	6.963-6.976, 4.289-4.300	unassigned
92	17,4-2	7.210-7.220, 8.452-8.595	phenylalanine, formate

No.* is the number shown in the R-STOCSY spectrum; SRV cluster IDs* are the numbers of SRV clusters data derived from the NMR spectra.

Supplementary Table S3: Differential metabolites identified from the OPLS-DA analysis of GS rats vs. CON rats (Figure 5A; ↑↑, ↓↓ denote p < 0.01; ↑,↓ denote p < 0.05)

Metabolites	Chemical shifts (ppm)	|r|	VIP	GS vs. CON*
LDL/VLDL	0.89(bar), 1.28(bar)	0.518	3.581	↓↓
3-hydroxybutyrate	1.19(d)	0.504	2.532	↓↓
alanine	1.49(d)	0.353	3.245	↓
arginine	1.64(m), 1.72(m)	0.355	1.407	↓
glutamine	2.13(m), 2.45(m)	0.366	1.638	↓
PUFA	2.81(bar)	0.791	3.458	↓↓
choline	3.20(s)	0.608	2.236	↓↓
PC	3.21(s)	0.359	1.651	↑↑
GPC	3.22(s)	0.539	2.216	↑↑
taurine	3.25(t), 3.40(t)	0.425	4.343	↑↑
glucose	3.23(m), 3.40(m),3.46(m), 3.53(dd), 3.72(m), 3.89(dd), 4.64(d), 5.22(d)	0.476	2.512	↑↑
threonine	1.33(d), 4.25(m)	0.391	1.261	↓↓
formate	8.46(s)	0.361	1.631	↑

Supplementary Table S4: Differential metabolites identified from the OPLS-DA analysis of LGD rats vs. CON rats (Figure 5B; ↑↑, ↓↓ denote $p < 0.01$; ↑,↓ denote $p < 0.05$)

Metabolites	Chemical shifts (ppm)	|r|	VIP	LGD vs. CON
LDL/VLDL	0.89(bar), 1.28(bar)	0.501	4.884	↓↓
lactate	1.33(d), 4.12(dd)	0.549	14.902	↑↑
arginine	1.64(m), 1.72(m)	0.347	1.489	↓
acetate	1.92(s)	0.404	2.625	↑↑
α-acid glycoprotein	2.04(s)	0.365	1.916	↑↑
glutamine	2.13(m), 2.45(m)	0.464	2.291	↓↓
PUFA	2.81(bar)	0.647	1.896	↓↓
PC	3.21(s)	0.314	1.475	↑
glycerol	3.54(dd), 3.64(dd)	0.324	1.184	↑
serine	3.95(m)	0.347	1.347	↑
tyrosine	6.89(d), 7.20(d)	0.378	1.328	↑↑
phenylalanine	7.32(d), 7.36(d), 7.41(t)	0.398	1.556	↑↑
xanthine	7.79(s)	0.329	1.424	↑
formate	8.46(s)	0.552	1.828	↑↑

Supplementary Table S5: Differential metabolites identified from the OPLS-DA analysis of HGD rats vs. CON rats (Figure 5C; ↑↑, ↓↓ denote $p < 0.01$; ↑,↓ denote $p < 0.05$)

Metabolites	Chemical shifts (ppm)	|r|	VIP	HGD vs. CON
LDL/VLDL	0.89(bar), 1.28(bar)	0.662	4.029	↓↓
lactate	1.33(d), 4.12(dd)	0.863	7.312	↑↑
alanine	1.48(d)	0.441	2.084	↓↓
arginine	1.64(m), 1.72(m)	0.599	1.340	↓↓
lysine	1.72(m), 189(m)	0.428	1.489	↑↑
α-acid glycoprotein	2.04(s)	0.603	1.341	↑↑
glutamine	2.13(m), 2.45(m)	0.433	1.167	↓↓
PUFA	2.81(bar)	0.847	1.839	↓↓
PC	3.21(s)	0.369	1.056	↑↑
taurine	3.25(t), 3.40(t)	0.302	1.332	↓
glycine	3.57(s)	0.402	1.054	↑↑
glucose	3.23(m), 3.40(m),3.46(m), 3.53(dd), 3.72(m), 3.89(dd), 4.64(d), 5.22(d)	0.314	2.014	↓
serine	3.95(m)	0.512	1.345	↑↑
tyrosine	6.89(d), 7.20(d)	0.323	1.378	↑
phenylalanine	7.32(d), 7.36(d), 7.41(t)	0.365	1.222	↑
histidine	7.01(s), 7.78(s)	0.312	1.146	↑
formate	8.46(s)	0.332	1.367	↑

Supplementary Table S6: Differential metabolites identified from the OPLS-DA analysis of GC rats vs. CON rats (Figure 5D; ↑↑, ↓↓ denote $p < 0.01$; ↑,↓ denote $p < 0.05$)

Metabolites	Chemical shifts (ppm)	|r|	VIP	GC vs. CON
LDL/VLDL	0.89(bar), 1.28(bar)	0.789	5.264	↓↓
3-hydroxybutyrate	1.19(d)	0.342	1.330	↑
lactate	1.33(d), 4.12(dd)	0.587	3.409	↑↑
arginine	1.64(m), 1.72(m)	0.303	1.185	↓
lysine	1.72(m), 189(m)	0.801	1.626	↑↑
α-acid glycoprotein	2.04(s)	0.581	3.911	↑↑
glutamine	2.13(m), 2.45(m)	0.434	1.394	↓↓
succinate	2.40(s)	0.761	2.249	↑↑
PUFA	2.81(bar)	0.515	1.351	↓↓
GPC	3.22(s)	0.754	2.290	↑↑
taurine	3.25(t), 3.40(t)	0.568	1.934	↓↓
glycine	3.57(s)	0.658	1.282	↑↑
glycerol	3.54(dd), 3.64(dd)	0.743	1.707	↑↑
glucose	3.23(m), 3.40(m),3.46(m), 3.53(dd), 3.72(m), 3.89(dd), 4.64(d), 5.22(d)	0.781	2.443	↓↓
serine	3.95(m)	0.392	1.402	↑↑
threonine	1.33(d), 4.25(m)	0.325	1.890	↑
tyrosine	6.89(d), 7.20(d)	0.653	1.532	↑↑
phenylalanine	7.32(d), 7.36(d), 7.41(t)	0.888	2.092	↑↑
xanthine	7.79(s)	0.409	1.597	↑↑
formate	8.46(s)	0.405	1.117	↑↑

Supplementary Table S7: Inter-metabolic correlations identified from the OR-STOCSY analysis of GS rats vs. CON rats (Figure 6A)

See Supplementary File 1

Supplementary Table S8: Inter-metabolite correlations identified from the OR-STOCSY analysis of LGD rats vs. CON rats (Figure 6B)

See Supplementary File 1

Supplementary Table S9: Inter-metabolite correlations identified from the OR-STOCSY analysis of HGD rats vs. CON rats (Figure 6C)

See Supplementary File 1

Supplementary Table S10: Inter-metabolite correlations identified from the OR-STOCSY analysis of GC rats vs. CON rats (Figure 6D)

See Supplementary File 1

Supplementary Table S11: Significant metabolites and enzymes extracted from the calculation of the shortest path lengths among the corrected metabolites, which were identified from the OR-STOCSY analysis of GS rats vs. CON rats (Figure 7A)

Metabolites	Enzymes
NADP+; Pyruvate;	EC 1.8.1.7
Acetyl-CoA; Glutamate;	EC 3.1.2.1
2-Oxoglutarate; Glucose	EC 1.4.1.2 EC 1.4.1.3
Acetate; Alanine; Lysine;	EC 6.3.1.2
Glutathione; Formate;	EC 2.6.1.2
Glutamine; Thiamin diphosphate;	EC 2.3.3.8
Phenylalanine;	EC 3.4.19.13
Acetaldehyde;	EC 1.2.4.2
Fructose; Fumarate;	EC 1.2.1.88
Leucine; Histidine;	EC 1.2.1.3
Proline; Citrate;	EC 1.1.1.2
Acetoacetate; Valine;	EC 3.2.1.10
Ethanolamine; 2-Oxoisocaproate;	EC 2.7.1.1 EC 2.7.1.4
Carnitine; Ethanol;	EC 3.7.1.5 EC 3.7.1.20
3-Hydroxybutanoate;	EC 2.6.1.6 EC 2.6.1.42
3-Methylhistidine;	EC 1.5.5.2
beta-D-Glucose 6-phosphate;	EC 4.1.3.4
2-Methylpropanoate;	EC 3.7.1.2
1-Pyrroline-5-carboxylate;	EC 2.3.1.12
beta-D-Fructose 6-phosphate;	EC 1.1.1.49 EC 1.1.1.363
S-Acetyldihydrolipoamide-E; PUFA	EC 1.2.4.1
	EC 5.3.1.9
	EC 1.2.4.4

Supplementary Table S12: Significant metabolites and enzymes extracted from the calculation of the shortest path lengths among the corrected metabolites, which were identified from the OR-STOCSY analysis of LGD rats vs. CON rats (Figure 7B)

Metabolites	Enzymes
α-acid glycoprotein;	EC 1.8.1.7
NADP+; Pyruvate	EC 3.1.2.1
Acetyl-CoA; Glutamate	EC 6.3.1.2
Glucose; Acetate	EC 2.6.1.2
Alanine; Lysine	EC 2.3.3.8
Glutathione; Formate	EC 3.4.19.13
Glutamine; Thiamin diphosphate	EC 1.2.1.88
Phenylalanine; Fructose	EC 3.2.1.20 EC 3.2.1.48
Fumarate; Leucine	EC 2.7.1.1 EC 2.7.1.4
Histidine; Proline	EC 3.7.1.5 EC 3.7.1.20
Citrate; Acetoacetate	EC 2.6.1.42 EC 2.6.1.67
Valine; 2-Oxoisocaproate	EC 1.5.5.2
Carnitine; Xanthine	EC 4.1.3.4
3-Hydroxybutanoate; 3-Methylhistidine	EC 1.1.1.30
beta-D-Glucose 6-phosphate	EC 3.7.1.6
2-Methylpropanoate;1-Pyrroline-5-carboxylate	EC 2.3.1.12
beta-D-Fructose 6-phosphate;	EC1.1.1.49 EC 1.1.1.363
S-Acetyldihydrolipoamide-E	EC 1.2.4.2
	EC 5.3.1.12
	EC 1.2.4.1

Supplementary Table S13: Significant metabolites and enzymes extracted from the calculation of the shortest path lengths among the corrected metabolites, which were identified from the OR-STOCSY analysis of HGD rats vs. CON rats (Figure 7C)

Metabolites	Enzymes
α-acid glycoprotein;	EC 2.2.1.6 EC 4.1.1.1
NADP+; Pyruvate;	EC 1.8.1.7
Acetyl-CoA; Glutamate;	EC 1.4.1.2 EC1.4.1.3
2-Oxoglutarate; Glucose;	EC 6.3.1.2
Alanine; Succinate;	EC 2.6.1.2
Lysine; Glutathione;	EC 2.3.3.9
Formate; Glutamine;	EC 3.4.19.13
Thiamin diphosphate;	EC 1.2.4.2
Phenylalanine; Fructose;	EC 1.2.1.12
Fumarate; Leucine;	EC 3.2.1.20 EC 3.2.1.48
Histidine; Proline;	EC 2.7.1.1 EC 2.7.1.4
Citrate; Acetoacetate;	EC 3.7.1.5 EC 3.7.1.20
Valine; 2-Oxoisocaproate;	EC 2.6.1.6 EC 2.6.1.67
Carnitine; Xanthine;	EC 1.5.5.2
3-Hydroxybutyrate;	EC 4.1.3.4
3-Methylhistidine; PUFA	EC 1.1.1.30
beta-D-Glucose 6-phosphate	EC 3.7.1.2
2-Methylpropanoate;	EC 2.3.1.12
1-Pyrroline-5-carboxylate;	EC 1.1.1.42
beta-D-Fructose 6-phosphate;	EC 1.2.4.4
S-Acetyldihydrolipoamide-E;	EC 5.3.1.9
	EC 1.2.4.4

Supplementary Table S14: Significant metabolites and enzymes extracted from the calculation of the shortest path lengths among the corrected metabolites, which were identified from the OR-STOCSY analysis of GC rats vs. CON rats (Figure 7D)

Metabolites	Enzymes
α-acid glycoprotein;	EC 2.2.1.6 EC 4.1.1.1
NADP+; Pyruvate;	EC 1.8.1.7
Acetyl-CoA; Glutamate;	EC 3.1.2.1
2-Oxoglutarate; Glucose;	EC 1.4.1.2 EC1.4.1.3
Acetate; Oxaloacetate;	EC 6.3.1.2
Glycine; Alanine;	EC 2.6.1.2
Succinate; Lysine;	EC 6.4.1.1
Glutathione; Formate;	EC 2.3.3.9
Glutamine; Serine;	EC 3.4.19.13
Thiamin diphosphate;	EC 2.6.1.45
Phenylalanine;	EC 1.2.4.2
Tyrosine; Acetaldehyde;	EC 1.1.1.27
Fructose; Fumarate;	EC 1.2.1.12
Leucine; Histidine;	EC 1.2.1.3 EC 1.2.1.5
Proline; Citrate;	EC1.1.1.2 EC 1.1.1.71
Acetoacetate; Valine;	EC 4.1.2.5
Lactate; Threonine;	EC 3.2.1.20 EC 3.2.1.48
2-Oxoisocaproate; Carnitine;	EC 2.7.1.1 EC 2.7.1.4
Isoleucine; Ethanol;	EC 3.7.1.5 EC 3.7.1.20
GPC; 3-Methyl-2-oxopentanoate;	EC 2.6.1.6 EC 2.6.1.67
3-Hydroxybutyrate;	EC 1.5.5.2
1-Methylhistidine;	EC 4.1.3.4
beta-D-Glucose 6-phosphate;	EC 1.1.1.30
2-Methylpropanoate; PUFA	EC 3.7.1.2
1-Pyrroline-5-carboxylate;	EC 1.4.3.2
beta-D-Fructose 6-phosphate;	EC 2.3.1.12
S-Acetyldihydrolipoamide-E;	EC 1.1.1.42
	EC 1.2.4.4
	EC 5.3.1.9
	EC 1.2.4.4
	EC 1.2.4.4
